# TMEM43-S358L mutation enhances NF-κB-TGFβ signal cascade in arrhythmogenic right ventricular dysplasia/cardiomyopathy

**DOI:** 10.1007/s13238-018-0563-2

**Published:** 2018-07-06

**Authors:** Guoxing Zheng, Changying Jiang, Yulin Li, Dandan Yang, Youcai Ma, Bing Zhang, Xuan Li, Pei Zhang, Xiaoyu Hu, Xueqiang Zhao, Jie Du, Xin Lin

**Affiliations:** 1 Tsinghua University-Peking University Joint Center for Life Sciences, Beijing, 100084 China; 2 Institute for Immunology, Department of Basic Medical Sciences, School of Medicine, Tsinghua University, Beijing, 100084 China; 3 The 7th Affiliated Hospital of Sun Yat-Sen University, Shenzhen, Guangdong 510275 China; 4 Department of Molecular and Cellular Oncology, The University of Texas, MD Anderson Cancer Center, Houston, TX 77030 USA; 5 Beijing Anzhen Hospital, Capital Medical University, The Key Laboratory of Remodeling-Related Cardiovascular Diseases, Ministry of Education, Beijing Collaborative Innovation Center for Cardiovascular Disorders, Beijing Institute of Heart, Lung & Blood Vessel Disease, Beijing, 100029 China

**Keywords:** TMEM43, ARVD, NF-κB, TGFβ, fibrosis, knock-in mouse

## Abstract

Arrhythmogenic right ventricular dysplasia/cardiomyopathy (ARVD/C) is a genetic cardiac muscle disease that accounts for approximately 30% sudden cardiac death in young adults. The Ser358Leu mutation of transmembrane protein 43 (TMEM43) was commonly identified in the patients of highly lethal and fully penetrant ARVD subtype, ARVD5. Here, we generated TMEM43 S358L mouse to explore the underlying mechanism. This mouse strain showed the classic pathologies of ARVD patients, including structural abnormalities and cardiac fibrofatty. TMEM43 S358L mutation led to hyper-activated nuclear factor κB (NF-κB) activation in heart tissues and primary cardiomyocyte cells. Importantly, this hyper activation of NF-κB directly drove the expression of pro-fibrotic gene, transforming growth factor beta (*TGFβ1*), and enhanced downstream signal, indicating that TMEM43 S358L mutation up-regulates NF-κB-TGFβ signal cascade during ARVD cardiac fibrosis. Our study partially reveals the regulatory mechanism of ARVD development.

**Electronic supplementary material:**

The online version of this article (10.1007/s13238-018-0563-2) contains supplementary material, which is available to authorized users.

## Introduction

Arrhythmogenic right ventricular dysplasia/cardiomyopathy (ARVD/C [MIM: 107970]) is a dominantly genetic disease of cardiac muscle with high risk of sudden cardiac death and heart failure. The prevalence of this disease in general population is about 1:5,000. The occurring rate in male is higher than that in female (Muthappan and Calkins, 2008; Pilichou et al., 2011). ARVD accounts for about 10%–30% of sudden cardiac death in young adults, particularly in young athletes (Capulzini et al., 2010). ARVD is characterized by progressively fibrofatty replacement of heart muscle mainly in the right ventricular myocardium, leading to ventricular arrhythmias and structural abnormalities. To date, 8 ARVD-associated genes have been identified. Among them, *TMEM43* is associated with ARVD5 [MIM: 604400], a highly lethal and fully penetrant ARVD subtype (Lombardi et al., 2011; Muthappan and Calkins, 2008).

Transmembrane protein 43 (TMEM43) is a four-transmembrane protein anchoring to endoplasmic reticulum and inner nuclear membrane (Schirmer et al., 2003). TMEM43 is highly conserved and presents in most species ranging from bacteria to human (Bengtsson and Otto, 2008; Merner et al., 2008). Analysis of the mRNA level suggested that *TMEM43* is widely distributed among a variety of human tissues including heart (Bengtsson and Otto, 2008). Previous studies have revealed TMEM43 pS358L mutation as a commonly genetic mutation in ARVD5 patients in several cohorts worldwide (Baskin et al., 2013; Christensen et al., 2011; Haywood et al., 2013; Merner et al., 2008; Milting et al., 2015). Although this mutation of TMEM43 is clearly associated with ARVD, the underlying molecular mechanism still remains to be determined.

Fibrosis, one of the key characters in ARVD/C, is predominantly featured with the excessive and abnormal deposition of extracellular matrix (ECM) components (Uitto and Kouba, 2000). The pro-fibrotic protein—transforming growth factor beta (TGFβ) and its target factor—connective tissue growth factor (CTGF) are considered as master switches to trigger the fibrotic program (Verrecchia and Mauviel, 2007). They initiate the SMAD signaling pathway that ultimately leads to the activation and proliferation of fibroblasts, which deposit extracellular matrix into the surrounding connective tissues. Under pathological conditions, cardiac fibroblasts are activated and transformed into myofibroblasts, which secrete exceeded ECM for cardiac fibrosis, resulting in cardiac stiffness, adverse myocardial remodeling and heart failure (Weber et al., 2013).

NF-κB is a master transcription factor that controls cell survival, cell proliferation, inflammatory response and cell cycle regulation (Hayden and Ghosh, 2008). The classical activation of NF-κB is mediated by pro-inflammatory cytokines (tumor necrosis factor alpha (TNFα), interleukin 1beta (IL-1β)), and varied receptors (toll-like receptors (TLRs), antigen receptors (AgR), receptor tyrosine kinases (RTKs) like epidermal growth factor receptor (EGFR) and human epidermal growth factor receptor type 2 (HER2), and G protein-coupled receptors (GPCRs)) (Hayden and Ghosh, 2004; Jiang and Lin, 2012; Pan and Lin, 2013). Upon receptor activation, several signaling intermediates are activated and lead to the activation of IκB kinase (IKK) complex (Karin and Ben-Neriah, 2000). Once activated, the IKK complex induces IκBα phosphorylation, leading to the ubiquitination and degradation of IκBα (Karin and Ben-Neriah, 2000). The degradation of IκBα releases NF-κB to shuttle from the cytoplasm into the nucleus, where NF-κB facilitates transcription of its target genes. Previous studies have demonstrated that T cell receptor (TCR) and B cell receptor (BCR)-induced NF-κB is mediated by the CARMA1-BCL10-MALT1 (CBM) complex (Hayden and Ghosh, 2004; Jiang and Lin, 2012). Similarly, several groups reported that GPCR-induced NF-κB activation is mediated by a non-hematopoietic CBM complex: containing CARD- and membrane-associated guanylate kinase-like domain-containing protein 3 (CARMA3), B-cell lymphoma protein 10 (BCL10) and mucosa-associated lymphoid tissue lymphoma translocation protein 1 (MALT1) (Grabiner et al., 2007; Klemm et al., 2007; McAllister-Lucas et al., 2007; Wang et al., 2007). The previous studies of our group have revealed that fully activation of IKK complex requires two independent signaling events: phosphorylation of IKKα/β and Lys63 (K63)-linked ubiquitination of NF-κB essential modulator (NEMO) (or IKKγ) (Shambharkar et al., 2007), and CBM complex plays an essential role in K63-linked ubiquitination of NEMO (Shambharkar et al., 2007). Based on a bi-molecule fluorescence complementation (BiFC) screen, we recently identified TMEM43 as a CARMA3-binding protein in EGFR-induced NF-κB activation (Jiang et al., 2016). Therefore, we hypothesize that TMEM43 may contribute to ARVD development by modulating NF-κB-dependent pathways.

To investigate the function of TMEM43 S358L mutation in ARVD development, we generated a mouse strain in which the DNA code of Ser358 was converted into that of Leu in *Tmem43* gene. We found that TMEM43-S358L mice display an ARVD-like phenotypes. TMEM43 S358L mutation leads to cardiac fibrosis through hyper-activation of NF-κB and up-regulation of TGFβ expression. These results highlight the functional impact of TMEM43 mutation in the development of ARVD disease and the underlying molecularly regulatory processes.

## Results

### TMEM43-S358L mutant mice display ARVD-like abnormalities

**Figure 1:**
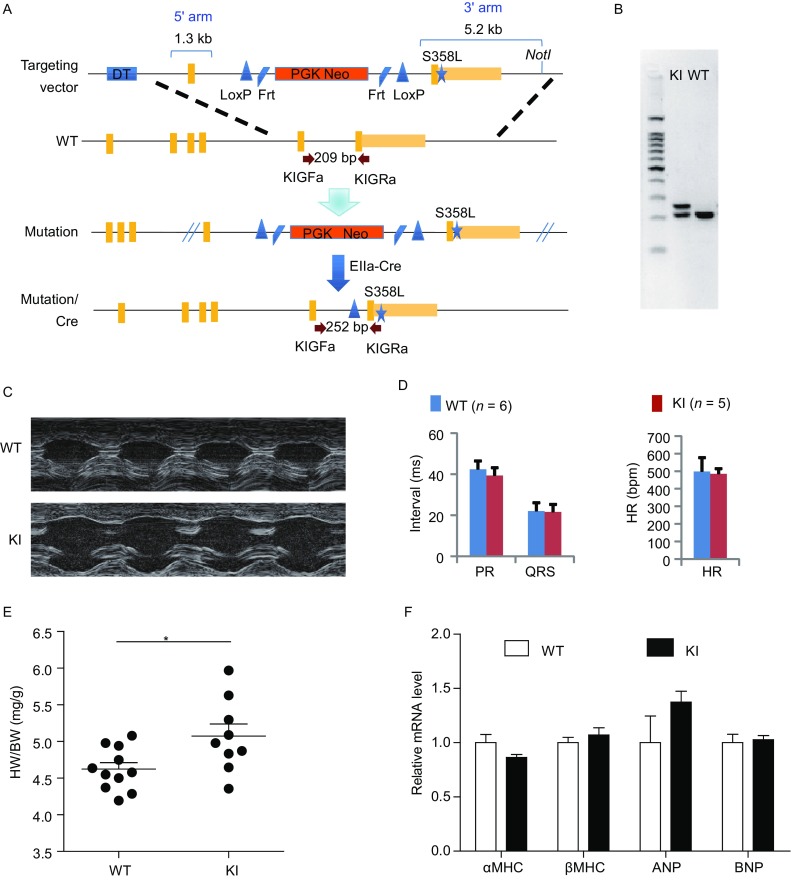
**Generation and characterization of ARVD TMEM43 mutation mice**. (A) The strategy of the construction of TMEM43 S358L mutation mouse. *DT* gene provided negative selection, while *Neo* gene provided positive selection of homo-recombination ES clones. Targeting vector carrying S358L point mutation was homo-recombinated with wild type genome through 1.3 kb 5′ arm and 5.2 kb 3′ arm. The obtained mutation mice were mated with whole body expression EIIα-Cre mice to remove the Neo selection cassette in the intron of *Tmem43* gene. (B) Genotyping of TMEM43 S358L knockin (KI) heterozygous mice had a 252 bp and 209 bp PCR product with primer sets of KIGFa and KIGRa, while wild type (WT) mice only had 209 bp product. DNA sequencing of the 252 bp PCR products verified the genomic mutation. (C) The represented figures in ultrasound analysis of littermate WT and TMEM43 mutation KI mice, indicating enlarged heart chambers in KI mice. (D) Electrocardiograph of littermate WT (*n* = 6) and KI (*n* = 5) mice. The heart rate (HR), PR interval and QRS interval were compared between the groups of WT and KI mice. Student’s *t*-test was used for statics analysis. (E) The heart weights and body weights of 8-week old male WT (*n* = 11) and KI (*n* = 9) mice were detected and the ratios of heart weights verse body weights were calculated. (F) Quantity PCR assay showed the mRNA level of the markers of cardiac hypertrophy in the hearts of WT (*n* = 5) and KI (*n* = 7) mice. Student’s *t*-test was used for statics analysis

Since TMEM43 S358L mutation is ubiquitously identified in ARVD5 patients, we generated TMEM43 S358L knock-in (KI) mice by homologous recombination-mediated genomic targeting (Fig. 1A). The genomic mutation in knock-in mice was verified via DNA sequencing of the PCR products from the amplification of mouse genome (Fig. 1B). ARVD is a dominantly genetic defect with much higher risk in young male persons. According to this feature of ARVD, we examined heterozygous TMEM43 S358L-KI mice of adult males that contain one allele of S358L mutation. Echocardiograph (Table 1 and Fig. 1C) of 8-week old mice showed that TMEM43 S358L mice have significantly higher level of left ventricle end-diastolic dimension (LVEDD) (*P*-value 0.0435) and lower level of posterior wall thickness in systole (PWTS) (*P*-value 0.0125). These results indicate enlarged cardiac chambers and thinner ventricular walls in TMEM43 S358L KI mice. Nevertheless, no obvious differences of electrocardiograph (ECG) were found in the KI mice (Fig. 1D). However, after tense running, 1 of 4 KI mice had ECG abnormality, but no arrhythmia was found in all 4 WT mice (Fig. S2A). In another experiment of tense exercises on running wheel of mice, we found one of 8 KI mice had sudden cardiac death, but no death was found in WT running group (6 mice) or in KI un-running group (5 mice) (Table S1). The ratio of heart weight to body weight was significantly higher in KI mouse group compared to control group (Fig. 1E). However, the expression of the markers of cardiac hypertrophy including myosin heavy chain alpha (*αMhc*, ID: 17888), myosin heavy chain beta (*βMhc*, ID: 140781), atrial natriuretic peptide (*Anp*, ID: 230899) and brain natriuretic peptide (*Bnp*, ID: 18158) were not significantly changed in the hearts of KI mice (Fig. 1F). These data suggest that KI mice may have slightly cardiac hypertrophy, similar to ARVD patients.

**Table 1:** Echocardiography of 8-week old male littermate WT and S358L mice

Parameter	WT	S358L	*P*-value
Number	8	5	
HR (bpm)	521.0 ± 43.7	528.8 ± 67.7	0.8429
LV mass index (mg)	89.6 ± 15.9	82.9 ± 15.5	0.5146
BW (g)	24.3 ± 2.1	24.1 ± 2.5	0.8886
LV mass/BW (mg/g)	3.66 ± 0.46	3.43 ± 0.49	0.4603
LVEDD (mm)	3.065 ± 0.424	3.525 ± 0.245	0.0435
LVESD (mm)	1.916 ± 0.318	2.024 ± 0.315	0.5987
IVSTD (mm)	1.098 ± 0.258	0.895 ± 0.081	0.0866
IVSTS (mm)	1.538 ± 0.276	1.346 ± 0.174	0.1866
PWTD (mm)	0.970 ± 0.146	0.787 ± 0.040	0.0125
PWTS (mm)	1.194 ± 0.117	1.210 ± 0.130	0.8512
FS (%)	37.6 ± 2.9	42.7 ± 6.6	0.2034
EDV (μL)	38.0 ± 12.5	52.2 ± 8.8	0.0521
ESV (μL)	12.0 ± 5.4	13.7 ± 5.7	0.6402
EF (%)	69.1 ± 4.0	74.2 ± 7.5	0.2631

Note: HR, heart rate; BW, body weight; LV, Left ventricular; IVSTD, interventricular septal thickness in diastole; IVSTS, interventricular septal thickness in systole; PWTD, posterior wall thickness in diastole; PWTS, posterior wall thickness in systole; LVEDD, LV end-diastolic dimension; LVESD, LV end-systolic dimension. FS, fractional shortening; EF, ejection fraction

**Figure 2:**
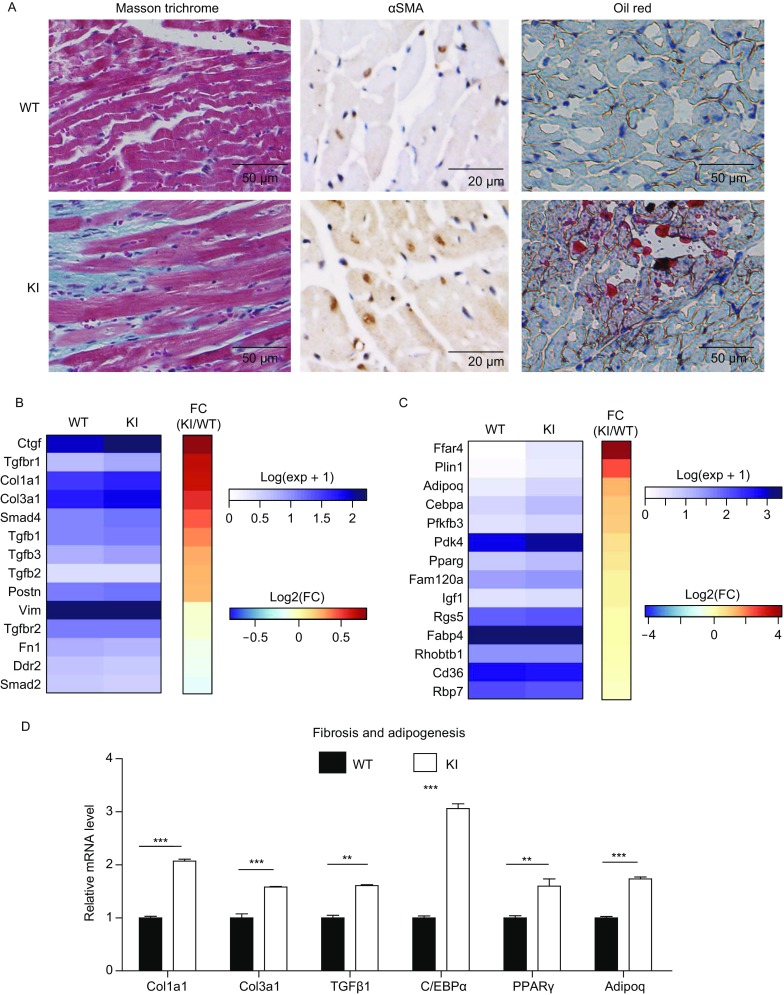
**TMEM43 mutation mice reproduced ARVD fibrofatty features**. (A) The hearts of 8-week old male WT (*n* = 17) and KI (*n* = 8) mice were fixed and sectioned in paraffin. The sections were subjected to masson’s trichrome staining (left panel). The blue staining indicated cardiac fibrosis region. The paraffin sections of the hearts of 8-week old male WT and KI mice were subjected to immunohistological staining via αSMA antibody (middle panel).The frozen sections of the hearts of 8-week old male WT (*n* = 3) and KI (*n* = 3) mice were stained with oil red (right panel). (B) The heat map showed the mRNA transcriptional level of the genes of cardiac fibrosis in RNA-seq assay of the hearts of WT (*n* = 4) and KI (*n* = 3) mice. (C) The heat map showed the mRNA level of the genes of cardiac adipogenesis in RNA-seq assay of the hearts of WT (*n* = 4) and KI (*n* = 3) mice. (D) Quantity PCR assay showed the mRNA level of the markers of cardiac fibrosis and adipogeneis in the hearts of WT (*n* = 4) and KI (*n* = 3) mice. *Gapdh* served as endogenous control. Student’s *t*-test was used for statics analysis. ** indicated *P*-value < 0.01, *** indicated *P*-value < 0.001

Next, we examined the fibrofatty features of ARVD in the hearts of KI mice with Masson’s trichrome staining, αSMA immunostaining and oil red staining. The fibrosis level (Fig. 2A, left panel and middle panel) and fat accumulation (Fig. 2A, right panel) were both elevated in the hearts of TMEM43 S358L KI mice. To explore the regulatory mechanism at molecular level, we carried out RNA-seq analysis to profile the transcript expression in the hearts of WT and KI mice. Compared to WT mice, heterozygous KI mice had higher expression (log2) of most of the markers of both fibrosis (Fig. 2B) and adipogenesis (Fig. 2C). We chose those genes whose expression changed higher than 4 folds in the hearts of WT and KI mice, and performed gene functional classification of DAVID (https://david.ncifcrf.gov). The only up-regulated signaling is peroxisome proliferator-activated receptor γ (PPARγ, ID: 19016) signaling pathway (Table 2), which is a major driver for adipogenesis. Our qPCR confirmation of RNA-seq result showed the significantly (all *P*-values < 0.05) increased expression of the markers of fibrosis such as *Col1a1*, *Col3a1* and *Tgf1*, as well as the markers of adipogenesis such as CEBPA encoding CCAAT/enhancer binding protein alpha (*Cebpa*, ID: 12606), *Pparg*, and adiponectin (*Adipoq*, ID: 11450) in the hearts of KI mice (Fig. 2D). Cardiac fibroblasts are transformed into myofibroblasts during cardiac fibrosis. We also found that the level of cardiac fibroblast significantly decreased (indicated by the expression of marker *Vim* and *Ddr2*), while the level of myofibroblast significantly increased (indicated by the expression of *αSma* and *Fn1*) in KI mice (Fig. S2C). To further verify the cardiac fibrosis of KI mice, primary cardiac fibroblasts and dermal fibroblasts isolated from postnatal day 3 pups of WT and KI mice were forced to transform into myofibrasts with TGFβ treatment. After treatment for 3 days or 7 days, the cells were immuno-fluorescence stained with αSMA antibody or collected for total RNA extraction and assayed with qPCR. The results indicated that TMEM43 ARVD mutant accelerates fibrosis progresses in both dermal fibroblasts (Fig. S4) and cardiac fibroblasts (Fig. S5). Taken together, these findings indicate that fibrofatty progression is accelerated in the hearts of TMEM43 S358L mice. Thus, TMEM43 S358L-KI mice partially recapitulate the pathological and histological phenotypes of ARVD patients.

**Table 2:** DAVID gene function classification of change genes in KI mouse hearts

Upregulated signaling	Downregulated signaling
PPAR signaling pathway	Metabolism of xenobiotics by cytochrome P450
	Drug metabolism
	Cytokine-cytokine receptor interaction
	Toll-like receptor signaling pathway
	Histidine metabolism
	MAPK signaling pathway
	Cysteine and methionine metabolism
	Prion diseases
	Chemokine signaling pathway
	Glutathione metabolism
	Synthesis and degradation of ketone bodies

### Hyper-activation of NF-κB by TMEM43 S358L mutant

**Figure 3:**
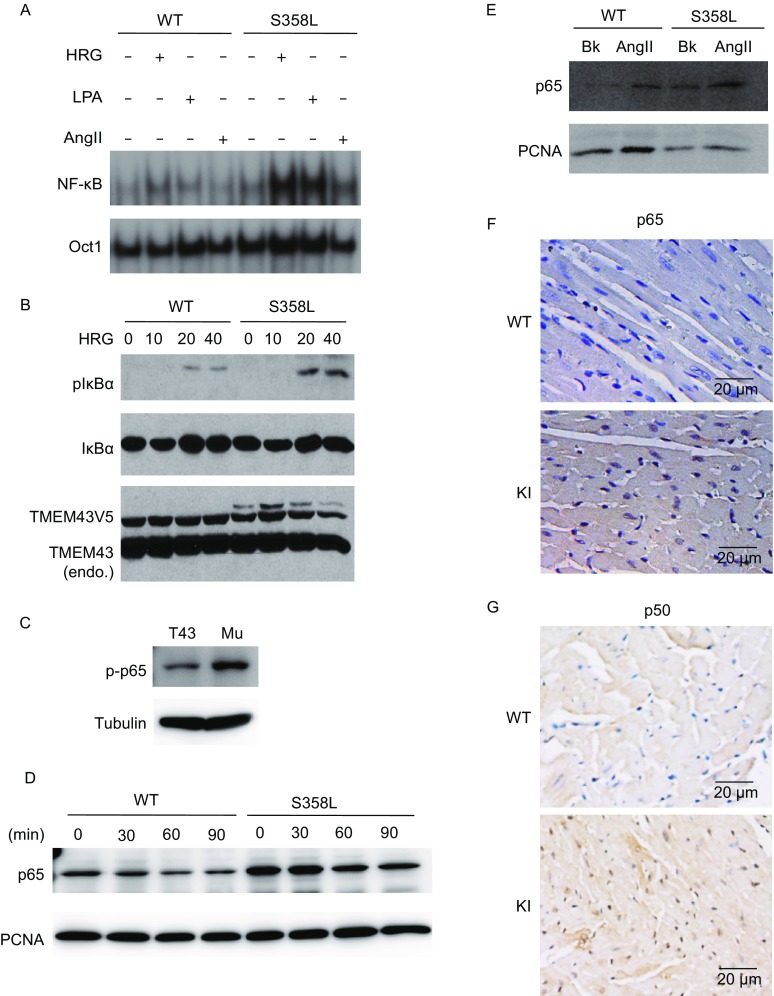
**TMEM43 ARVD mutant enhanced NF-κB activity**. (A) Primary human cardiomyocytes stably expressing V5-tagged TMEM43 WT or S358L mutant were serum starved for overnight and stimulated with HRG (50 ng/mL), LPA (10 μmol/L) or Ang II (1 μmol/L) for 60 min. Nuclear extracts were isolated and subjected to EMSA analysis with P^32^ labelled nucleotide probe of p65 binding substrate. OCT-1 served as a loading control. (B) Cardiomyocytes stably expressing vector or V5-tagged TMEM43 WT or S358L mutant were serum starved for overnight and stimulated with 50 ng/mL of HRG for the indicated time. The cell lysates were subjected to immunoblotting of the indicated antibodies. (C) The total protein of A549 cells stably expressing TMEM43 WT or S358L mutant were collected and lysed. The cell lysates were subjected to immunoblotting of the indicated antibodies. (D) A549 cells stably expressing TMEM43 WT or S358L mutant were serum starved for overnight and stimulated with or without AngII (4 μmol/L) for the indicated time. The nuclear extracts were subjected to immunoblotting of p65 antibody and PCNA antibody. (E) The MEF cells derived from WT and KI mice were serum starved for overnight and stimulated with or without AngII (10 μmol/L) for 60 min. The nuclear extracts were collected and subjected to immunoblotting of p65 antibody and control PCNA antibody. (F) The paraffin sections of the hearts of 8-week old male WT and KI mice were performed immunohistological staining via p65 antibody. (G) The paraffin sections of the hearts of 8-week old male WT and KI mice were performed immunohistological staining via p50 antibody

Our recent study revealed that TMEM43 interacts with CARMA3 and mediates EGFR-induced NF-κB activation (Jiang et al., 2016). Since TMEM43 S358L mutation is a cause of the cardiac muscle disease of ARVD5 (Milting et al., 2015), we hypothesized that TMEM43 S358L mutation might alter its effect on NF-κB activation. To determine the relevant function of TMEM43 S358L mutation in human cells, we stably overexpressed the proteins of WT TMEM43 or its S358L mutant in human primary cardiomyocytes. Resulted cells were stimulated with EGFR ligand HRG, GPCR ligand, lysophosphatidic acid (LPA) or angiotensin II (AngII). The binding ability of Nuclear NF-κB (p65) to its transcriptional binding sites was examined by electrophoretic mobility shift assay (EMSA) to determine the activation of NF-κB. Compared to TMEM43 WT cells, we observed higher binding ability of p65 (Fig. 3A) in TMEM43 S358L-expressing cardiomyocytes. To confirm this observation, the same cells were stimulated with HRG for the indicated time and subjected to immunoblotting of phosphorylated IκBα, an indication of the activity of NF-κB. We detected higher amount of phosphorylated IκBα in TMEM43 S358L-expressing cardiomyocytes (Fig. 3B). Higher amount of phosphorylated p65, another indication of the activity of NF-κB, was also found in TMEM43 S358L-expressing cells (Fig. 3C). Similarly, upon AngII stimulation with the indicated time, the nuclear translocation of p65 significantly increased in cells expressing TMEM43 S358L mutant (Fig. 3D). We also examined nuclear translocation of NF-κB (p65) in mouse embryonic fibroblast cells (MEFs) derived from WT and heterozygous TMEM43 S358L KI mice, and found that the nuclear translocation of p65 was significantlyenhanced in TMEM43 S358L MEFs under the conditions with or without AngII stimulation (Fig. 3E). Upon immunohistochemistry staining of NF-κB components, p65 and p50, on paraffin sections of heart tissues, KI mice had more cells with nuclear staining of p65 and p50 than WT mice (Fig. 3F and 3G). Together, these results reveal that TMEM43 S358L mutation enhanced NF-κB activation *in vitro* and *in vivo*.

### Inflammation was not increased in TMEM43-S358L mutant mice

**Figure 4:**
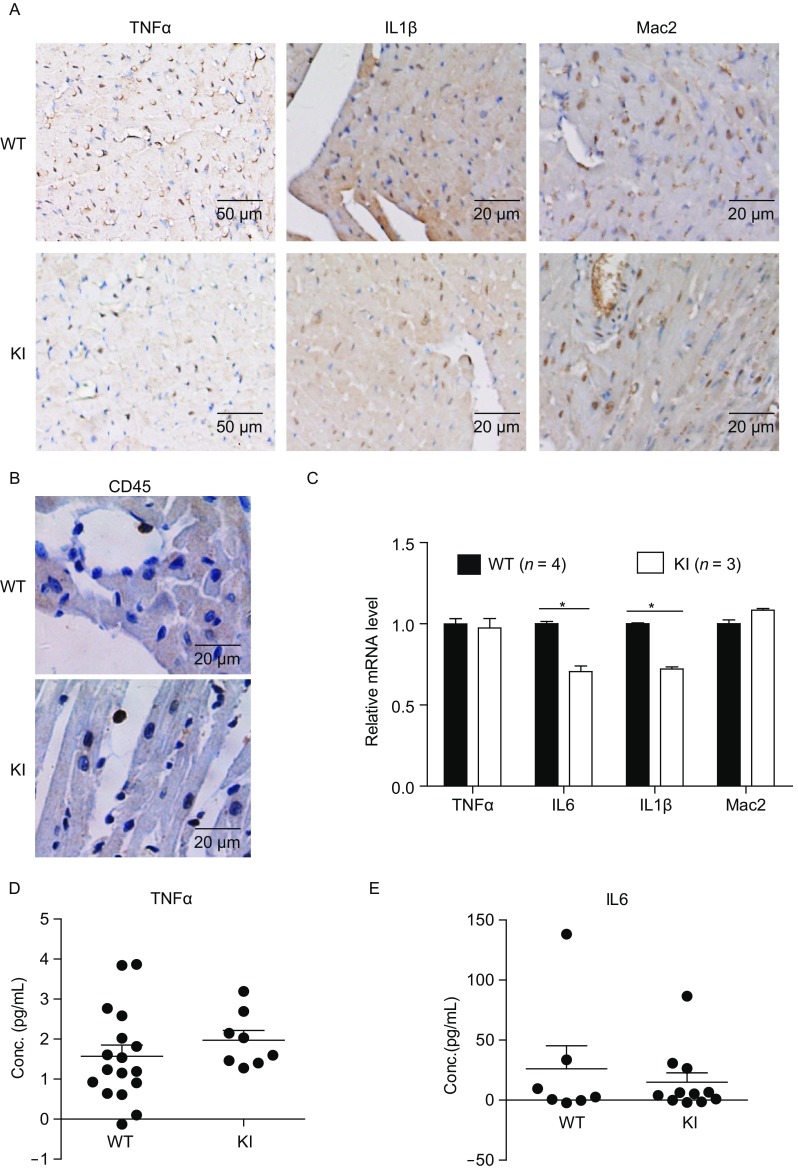
**The inflammation did not increase in TMEM43 mutation mice**. (A) The paraffin sections of the hearts of 8-week old male WT and KI mice were performed immunohistological staining via pro-inflammatory cytokine TNFα, IL1β antibody and macrophage marker MAC2 antibody. (B) The paraffin sections of the hearts of 8-week old male WT and KI mice were performed immunohistological staining via CD45 antibody. (C) Quantity PCR assay showed the mRNA level of pro-inflammatory cytokines in the hearts of WT (*n* = 4) and KI (*n* = 3) mice. Student’s t-test was used for statics analysis. The star * indicated *P*-value < 0.05 in Student’s *t*-test. (D) The level of TNFα cytokine in the serum of 8-week old male WT (*n* = 17) and KI (*n* = 8) mice were analyzed via enzyme linked immunosorbent assay (ELISA). (E) The level of IL6 cytokine in the serum of 8-week old male WT (*n* = 7) and KI (*n* = 11) mice was analyzed via ELISA

Since one of the critical roles of NF-κB is to control the expression of pro-inflammatory cytokines, we investigated the level of pro-inflammatory cytokines in the cardiac tissues of WT and heterozygous TMEM43 S358L KI mice. Surprisingly, the immunohistochemistry of pro-inflammatory cytokine TNFα (ID: 21926), IL1β (ID: 16176), macrophage marker MAC2 (ID: 16854) (Fig. 4A) and the common leukocyte marker CD45 (ID: 19264) (Fig. 4B) revealed lower or no changed level of inflammation in heterozygous TMEM43 S358L KI mice. The qPCR analysis of cardiac mRNA showed that the expression of inflammatory cytokines such as IL6 (ID: 16193) and IL1β in the hearts of KI mice was slightly lower than that of WT mice (Fig. 4C). This observation is consistent to gene functional classification of DAVID in RNA-seq, which highlights that several signaling down-regulated in the hearts of the KI mice were pro-inflammatory pathways including Toll-like receptor signaling pathway, p38 mitogen-activated protein kinase (MAPK) signaling pathway and chemokine signaling pathway (Table 2). To further confirm the observation, the level of cytokines in murine serum was analyzed via enzyme linked immunosorbent assay (ELISA). No significant differences of the expression of TNFα and IL6 were found between WT and KI mice (Fig. 4D and 4E). Thus, the NF-κB signal-related inflammatory response is not involved in the TMEM43 mutation-associated ARVD pathology.

### NF-κB1 synergizes with p65 to directly regulate pro-fibrotic gene *Tgfβ1* expression and enhance TGFβ signaling activity

**Figure 5:**
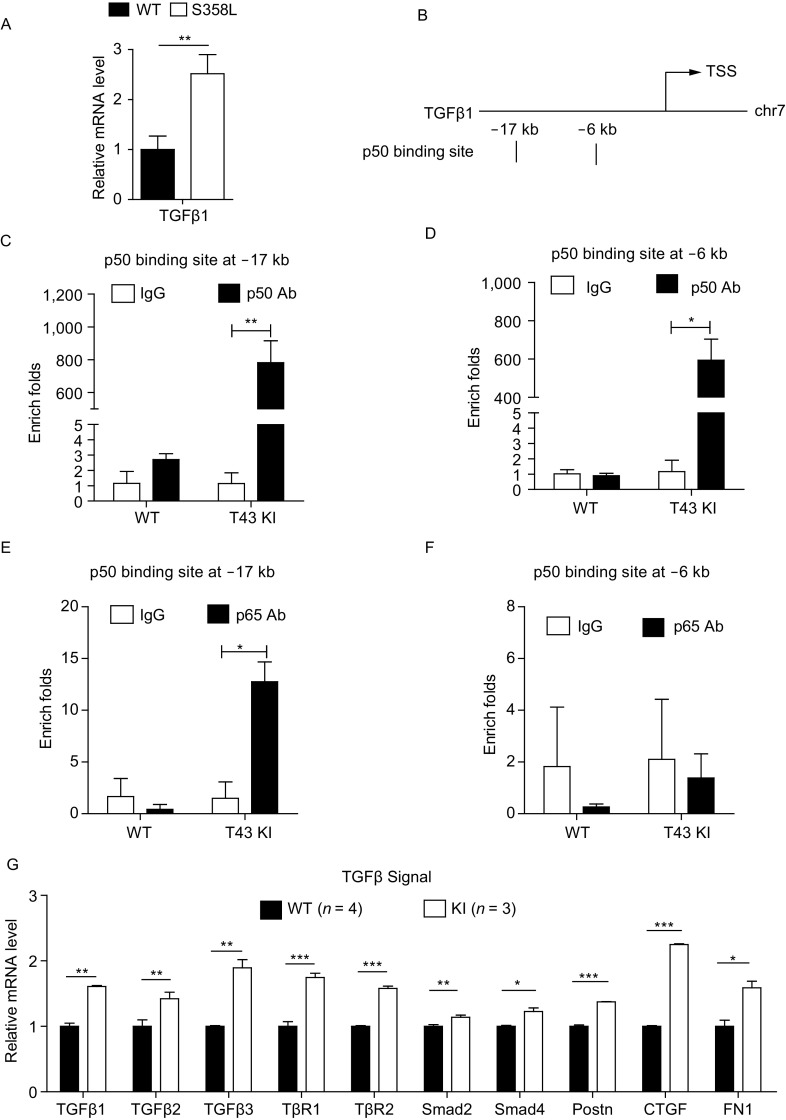
**NF-κB1 synergized with p65 to directly control** ***Tgfβ1*** **expression, thus enhanced TGFβ signaling activity**. (A) mRNA isolated from cardiomyocytes stably expressing V5-tagged TMEM43 WT or S358L mutant were measured by quantitative real-time PCR. Student’s *t*-test was used for statics analysis. Two stars ** indicated *P*-value < 0.01 in Student’s *t*-test. (B) Qiagen website predicted the NF-κB1 (p50) binding sites on the promoter of *Tgfβ1* at mouse genome. (C) The hearts of 8-week old male WT and KI mice were subjected to Chromatin Immunoprecipitation (ChIP) assay via IgG and p50 antibodies. The enriched folds of antibody binding were assayed by qPCR at −17 kb binding site. (D) The enriched folds of antibody binding were assayed by qPCR on −6 kb binding site. (E) The hearts of 8-week old male WT and KI mice were subjected to Chromatin Immunoprecipitation (ChIP) assay via IgG and p65 antibodies. The enriched folds of antibody binding were assayed by qPCR on −17 kb binding site. (F) The enriched folds of antibody binding were assayed by qPCR on −6 kb binding site. Student’s *t*-test was used for statics analysis. One star * indicated *P*-value < 0.05, two stars ** indicated *P*-value < 0.01 in Student’s *t*-test. (G) Quantity PCR assay showed the mRNA level of genes of TGFβ signal in the hearts of WT (*n* = 4) and KI mice (*n* = 3). *Gapdh* served as endogenous control. Student’s *t*-test was used for statics analysis. * indicated *P*-value < 0.05, ** indicated *P*-value < 0.01, *** indicated *P*-value < 0.001

**Figure 6:**
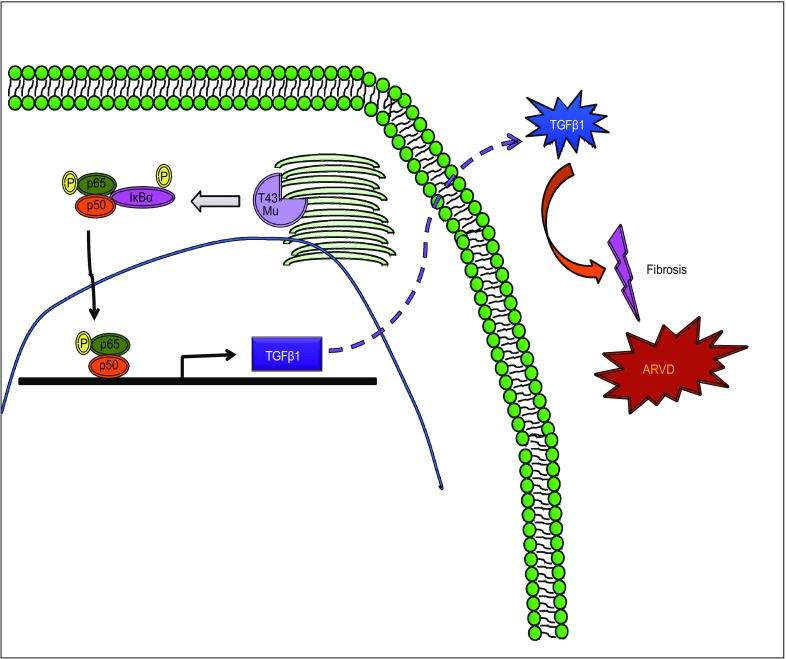
**TMEM43 S358L mutant enhanced cardiac fibrosis during ARVD development**. TMEM43 S358L mutation hyper-activates NF-κB signal, which further induces *Tgfβ1* expression and enhances TGFβ signaling pathway. Increasing activation of TGFβ signaling promotes fibrosis during ARVD development

Since inflammation appears not the cause of TMEM43 mutation-associated ARVD pathogenesis, we hypothesized that other pathways induced by TMEM43 S358L mutation may contribute to the pathogenesis. We noticed that the mRNA level of pro-fibrotic gene *TGFβ1* was significantly elevated in primary cardiomyocytes of TMEM43 S358L mutant (Fig. 5A). Therefore, we searched the binding sites of transcription factors on mouse *Tgfβ1* promoter. NF-κB1 (p50, ID: 18033) ranks at top 6 of the transcription factors that are predicted to bind to the promoter regions of both human and mouse *Tgfβ1* (ID: 21803) by Qiagen database (http://www.sabiosciences.com). In accordance with the predicted binding sites (Fig. 5B), chromatin immunoprecipitation (ChIP) assay was performed and the enrich folds of both binding sites at −17 kb (Fig. 5C) and −6 kb (Fig. 5D) in TGFβ1 promoter were detected. Immunoprecipitation (IP) of p50 antibody enriched hundreds of folds at both sites in the heart tissues of TMEM43 S358L KI mice, but not in that of littermate WT mice. This result suggests that p50 binds to both −17 kb and −6 kb binding sites in TMEM43 S358L KI mice, but not in WT ones. Since p50 can form homo-dimer to execute repression function or form heterodimer with another NF-κB subunit p65 (ID: 19697) to execute activation function. To distinguish these two possibilities, we performed the ChIP assay by IP p65 from the lysates of mouse hearts, and determined the enrichment of predicted p50-binding sites in the promoter of *Tgfβ1* in the immunoprecipitated mixtures by qPCR assay. Our results showed that p65 could enrich the p50 binding site at −17 kb of *Tgfβ1* promoter (Fig. 5E), but not the binding site at −6 kb (Fig. 5F) in the heart tissues of TMEM43 S358L KI mice. Together, these results suggest that p50 synergizes with p65 and directly binds to the predicted p50-binding site at −17 kb of *Tgfβ1* promoter, thereby promoting the expression of *Tgfβ1* in the hearts of TMEM43 S358L KI mice.

There are several predicted p65 binding sites in mouse *Tgfβ1* promoter (Fig. S1A). However, the ChIP assay revealed that all of the predicted binding sites including the binding sites at −11 kb, −6 kb, 1 kb and 3 kb (Fig. S1B–E) were not enriched by p65 antibodies in the heart tissues of TMEM43 S358L mice. These results suggest that p65 do not bind to these predicted binding sites in *Tgfβ* promoter.

We then investigated the activation of TGFβ signal in the hearts of TMEM43 S358L KI mice. The qPCR analysis of heart tissues (Fig. 5G) showed that the mRNA level of the components in TGFβ1 signaling such as *Tgfβ1*, *Tgfβ3* (ID: 21809), *Smad2* (ID: 17126), periostin (*Postn*, ID: 50706), *Ctgf* (ID: 14219) and fibronectin (*Fn1*, ID: 14268) were significantly elevated in TMEM43 S358L KI mice. The level of nuclear pSmad is one indicator of the activity of TGFβ signal. Western blot analysis of nuclear pSmad2 also verified the increasing activation of TGFβ signaling in TMEM43 S358L cells when stimulated with or without Angiotensin II for the indicated time (Fig. S1F). Both of the immunofluorescence staining and qPCR analysis indicated that the treatment with inhibitors of TGFβ signal—LY2109761 reversed TMEM43 S358L induced fibrosis progresses in dermal fibroblasts (Fig. S6). These results indicate that TMEM43 S358L mutation hyper-activates NF-κB signal, which subsequently induces the expression of *Tgfβ1* and enhances the activation of TGFβ signaling.

## Discussion

Previous studies all utilized transgenic mutant mice or genetic knockout mice to study the molecular mechanism in ARVD. In this study, we generated a mouse model carrying the genetic mutation found in human patients. Our TMEM43 S358L KI mice developed cardiac fibrosis and adipogenesis, similar to ARVD patients. Their cardiac morphology changed like human ARVD. Transcriptome assay and qPCR analysis of the markers of fibrosis and adipogenesis revealed the higher level of both fibrosis and adipocyte formation in the hearts of KI mice. When dissecting the involved signal pathways, we find that the TMEM43 S358L mutation hyper-activated NF-κB signal as expected. However, this activation does not promote typically inflammatory responses. Instead, it induces another signal—TGFβ in fibrosis progress. Together, our studies provide the genetic evidences that TMEM43 S358L mutation contributes to the development of ARVD pathogenesis, and reveal a novel mechanism by which TMEM43 S358L mutation induces the development of ARVD.

The difficulty in the analysis of mouse echocardiograph and electrocardiograph is due to the much higher beating rate and smaller size of their hearts compared to human ones (Cranefield, 1975). Since the dilation of cardiac chambers in ARVD patient is almost limited in right ventricle (Muthappan and Calkins, 2008), this may explain that echocardiograph of our KI mice does not show very obviously structural and functional abnormalities in the left ventricle. However, left ventricle enlargement (LVE), the most prevalent feature of ARVD5 (Hodgkinson et al., 2013; Merner et al., 2008) was also observed in the KI mice. ARVD5 patients have significantly extended QRS duration (Merner et al., 2008), while we did not observe this phenotype in our KI mice. The much highly beating rate of mouse hearts may explain the difficulty in detection of cardiac electrical disorders. Of note, the histological results revealed increasing fibrosis infiltration and adipocyte accumulation in *Tmem43* KI mouse hearts, which reemerged the histological characters of ARVD5 in human patients (Hodgkinson et al., 2013; Merner et al., 2008). These findings suggest that the S358L mutation of TMEM43 is deleterious.

It has been reported that TGFβ1 and NF-κB were both involved in rat liver fibrosis (Zhou et al., 2015) and liver cirrhosis, in which the expression of TGFβ and the nuclear translocation of NF-κB were both required (Chavez et al., 2012). The same observation was found in kidneys (Zhang et al., 2015). Similarly, our studies reveal that TMEM43 S358L mutation strengthens NF-κB signaling and enhances *Tgfβ* mRNA expression, thus leading to fibrosis in mouse hearts. It has been shown that TGFβ is the master regulator of fibrosis in various tissues (Meng et al., 2016; Xu et al., 2016), but how NF-κB controls *TGF* expression still remains unclear. Here we show that NF-κB directly regulates *Tgf* transcription through the binding of *Tgfβ* promoter with p65/p50 heterodimer. The expression of *Tgfβ* subsequently enhances fibrosis in the heart of TMEM43 S358L KI mice (Fig. 6). Thus, TMEM43 S358L mutation enhances the activation of NF-κB and TGFβ signaling, which results in the increasing fibrosis in the heart.

The increasing expression of NF-κB targets of pro-inflammatory factors like IL-6 and IL-1β were observed in the hearts of cardiac conditional knockout plakoglobin (PG) ARVD mouse model (Li et al., 2011). Also in the serum of ARVD patients, the expression of IL-6, IL-1β and TNFα was significantly increased (Campian et al., 2010). It has been reported that the level of several pro-inflammatory factors significantly increased in the serum of ARVD patients and the low concentration of inflammatory factors was enough to drive the alteration of cellular location of Plakoglobin (JUP), an ARVD-inducing desmosome protein (Asimaki et al., 2011). Consistently, the hearts of severe ARVD patients had infiltration of lymphocytes (Campuzano et al., 2012). Therefore, we expected that NF-κB-mediated inflammation plays important roles in ARVD. Surprisingly, we found that the inflammatory level was not elevated in the hearts of TMEM43 S358L knock-in mice. This lack of inflammation may be due to the up-regulated PPARγ pathway in these mice, since PPARγ has a strong anti-inflammatory effect (Bassaganya-Riera et al., 2010; Park, 2016; Wada and Kamisaki, 2010). Another possibility is that unlike macrophage, monocyte, and dendritic cells, which have robust inflammatory response to NF-κB signal, cardiomyocytes may have weak inflammatory responses to NF-κB activation. It has been reported that the exposure of lipopolysaccharide, which stimulates NF-κB signal, to cardiomyocyte did not produce increasing expression of cytokines including TNFα, IL6 and IL1β (Maass et al., 2005; Niederbichler et al., 2006).

TMEM43 S358L mice partially reproduce the features of ARVD, and can be utilized in future drug detections. Importantly, our studies suggest that TMEM43 S358L mutation contributes to cardiac fibrosis through NF-κB-TGFβ signal cascade during ARVD progress. The mechanism of fat accumulation in the hearts of TMEM43 S358L mice needs further investigations. This work provides some novel drug targets and ultimately will benefit the management and therapy of ARVD.

## Materials and methods

### Generation of TMEM43 S358L KI mice

A 1.3 kb genomic fragment and a 5.2 kb genomic fragment of *Tmem43* were PCR amplified from the BAC clone RP24-335A15 of C57BL/6 male mouse. The point mutated (TCC->TTA, S358L) in 5.2 kb fragment was constructed via nest PCR. The 1.3 kb fragment and the 5.2 kb mutation fragment were then sub-cloned into KO II vector for generation of a targeting vector (Fig. 1A). The vector was linearized at a unique *Not*I site and electroporated into ES cells of 129/B6 hybrid. Positive clones were identified by PCR amplification of both homo-recombination arms in genome. Targeted ES cells were micro-injected into C57BL/6 blastocysts to generate chimeras mice. Mouse chimeras were bred to wild type mice of 129 background. The obtained mutation mice were mated with mice of whole body expression EIIα-Cre (Lakso et al., 1996) to remove the selection cassette of *Neo* in the intron of *Tmem43* (Fig. 1A). The *Tmem43* knockin (KI) mice were genotyped by PCR (2*EasyTaq PCR SuperMix, Transgen, Cat# AS111-14) and the obtained PCR products were DNA sequenced to confirm the mutation in genome. The wild type and heterozygous KI mice of 8-week old male littermate were analyzed in the following experiments. The body weights of mice were detected. Mice were sacrificed, the hearts were dissected and weighted. The ratios of heart weight (mg) to body weight (g) were calculated and compared between WT and KI mouse groups. WT and S358L-KI mice of 2- or 6-month old male were placed on a running wheel to perform intense running at an intensity of 1 h running with 10 min rest for 5 h per day, and continuously exercised for 7 days.

### Echocardiographic analysis of mouse hearts

Mice were anesthetized with isoflurane. Echocardiography was performed utilizing the high-resolution ultrasound system of VisualSonics Vevo 770 with a 30 MHz transducer (Visual sonic, Toronto, Canada). The hearts were 2D imaged in the view of parasternal short-axis. Echocardiographic measurements were performed on M-mode in triplicates from more than 5 mice per group.

### Electrocardiographic analysis

Mice were anesthetized and placed on a warming pad. The electrocardiography (ECG) of Lead II surface was carried out via a pair of electrodes that were connected to a bio-amplifier. At least 6 single cycles of ECG per mouse were analyzed to obtain the average PR interval and QRS interval.

### Oil red staining

Hearts from wild type and *Tmem43* KI mice were collected, embedded in OPT and frozen at −20 °C. The blocks of frozen heart were cut into 8 μm sections at −20 °C. The frozen myocardial sections were fixed with 10% formalin in PBS, washed with distilled water, and placed in 100% propylene glycol for 3 min. Slides were stained with Oil Red O solution (0.5% *w*/*v* in propylene glycol) for 20 min at 37 °C, then placed in 85% propylene glycol for 3 min, rinsed with distilled water, and counterstained with modified Mayer’s hematoxylin. The slides were covered with 10% glycerol in PBS and imaged under light microscope.

### Histological analysis

Hearts, kidneys and spleens from wild type and *Tmem43* KI mice were collected and fixed with 4% paraformaldehyde overnight at 4 °C. Hearts and kidneys were embedded in paraffin, and cut into 4 μm sections. The sections were stained with Masson’s Trichrome staining (Polysciences Inc.) following the manufacturer’s protocol. The paraffin sections of mouse hearts or spleens were immunostained with specific antibodies: p65, p50, IL-1β, MAC2, TNFα, CD45 (Santa Cruz) and αSMA (Abcam, Cat# ab5694).

### RNA isolation, quantity PCR and RNA-seq

Total RNA from mouse hearts or cardiomyocyte cells were extracted via Trizol Reagent (Ambion, life technology). RNA was reversely transcribed to cDNA with GoScript Reverse Transcriptase (Promega) following the manufacturer’s instructions. Quantitative real-time PCR was performed in triplicate determinants using SYBR green (Genestar, Cat#A313-10) detection on a thermal cycler of ABI7500. The relative mRNA levels of specific genes (*Anp*, *Bnp*, *Col1α1*, *Col3α1*, *Tgfβ1*, *Cebpa*, *Pparg*, *Adipoq*, *Tgβ2*, *Tgfβ3*, *TβR1*, *TβR2*, *Smad2*, *Smad4*, *Postn*, *Ctgf*, *Fn1*, *Tnfα*, *Il6*, *Il1β*, *Mac2*, *αSma*, *Ddr2*, *Vim*, *Jup*, *Pkp2*, *Dsp*, *Ccpg* and *Plin*) were normalized to glyceraldehyde-3-phosphate dehydrogenase (*Gapdh*).

For RNA-seq, a total amount of 1.5 μg RNA per sample was used as input material for the preparations of RNA libraries. Sequencing libraries were generated using NEBNext®UltraTMRNA Library Prep Kit for Illumina® (NEB, USA) following manufacturer’s recommendations and index codes were added to attribute sequences to each sample. The clustering of the index-coded samples was performed on a cBot Cluster Generation System using HiSeq 4000 PE Cluster Kit (Illumia) according to the manufacturer’s instructions. After cluster generation, the library preparations were sequenced on an Illumina Hiseq 4000 platform and 150 bp paired-end reads were generated. The expression values are presented as heat map.

### Cell lines

Human primary cardiomyocytes: pcDNA3.1/V5-His TOPO-*TMEM43* and pcDNA3.1/V5-His TOPO-*TMEM43 S358L* were kindly provided by Dr. Luiz Bengtsson (Berlin, German). V5-tagged *TMEM43* WT or S358L were PCR amplified and subcloned into the lentiviral vector pMX-IRES-GFP. Lentiviruses carrying TMEM43 WT or S358L were produced in HEK293T cells by transfection of either lentiviral plasmid together with the packaging plasmids, and subsequently used to infect the primary cardiomyocytes of human, which were purchased from Celprogen (Cat# 36044-15). The infected cardiomyocytes were subjected to puromycin selection at 1 μg/mL for 3 days. The expression levels of WT or *S358L TMEM43* in these resulting stable cells were validated by Western blot.

A549 cell lines: A549 cells were cultured with DMEM containing 10% FBS, at 37 °C, 5% CO_2_. A549 cells were infected with lentivirus carrying the p2k7 vector, HA-Flag-TMEM43 and HA-Flag-TMEM43 S358L mutant, and treated with blasticidin (BSD) for 7 days for selection of positive clones.

MEF cells: Embryos at stage of E10.5–E13.5 were collected. The limbs, heads and visceral tissues were all removed. The remaining bodies were minced into small pieces and digested with 0.25% trypsin solution at 37 °C, 5% CO_2_ for 10 min. The cells were isolated from tissues by vigorously pipetting, and the trypsin was neutralized with DMEM containing 10% FBS. After incubation at 37°C, 5% CO_2_ for 24–48 h until 100% confluence, the cells were harvested and marked as passage 0. MEF cells of passage 3–5 were used in the experiments.

Dermal fibroblasts: Mouse pups at neonatal day 1–3 were sacrificed. The skin was cut and removed from the body. The obtained skins were sunk in 2.5% trypsin (no EDTA) at 4 °C overnight for digestion. After digestion, the skins were transferred into PBS and tore carefully with forceps to detach the dermal layer from epidermal layers. The dermal layers were minced into 1 × 1 mm^3^ small pieces, and incubated in 1,000 U/mL collagenase solution at 37 °C with stirring at 1,200 rpm for 1–2 h. The digestion was stopped via adding FBS and filtered with strainers of 70 μm nylon meshes to remove debris. The product was collected and plated at 10 cm dishes. The dermal fibroblasts were passaged until 100% confluence. The cells of passage 3–5 were used in the experiments. Dermal fibroblasts isolated from WT and Tmem43 KI pups of p3–4 day old were treated with 10 ng/mL TGFβ in DMEM for 3 days or 7 days and subjected to immunofluorescence (IF) staining with αSMA antibody or RNA isolation. Dermal fibroblasts isolated from WT and Tmem43 KI pups of p3–4 day old were treated or untreated with 10 μmol/L TGFβ inhibitor—LY2109761 (Selleck, Cat#S2704) for 3 days and subjected to immunofluorescence (IF) staining with αSMA antibody. Dermal fibroblasts were treated or untreated with LY2109761 for 3 days and the total RNA of cells was isolated for qPCR analysis.

Cardiac fibroblasts: Mouse pups at neonatal day 1–3 were sacrificed. The hearts were collected and digested at 37 °C for 10 min in 80 mL Ads Buffer (20 mmol/L HEPES, 120 mmol/L NaCl, 1 mmol/L NaH_2_PO_4_, 5.5 mmol/L glucose, 5.4 mmol/L KCl, 0.8 mmol/L MgSO_4_ (pH 7.3–7.4)) with 55 mg pancreatin and 28.5 mg collagenase II. The first time digestion was discarded. The digestion was repeated for 6–8 times and the cells were harvested. The cells were next plated in dishes and incubated at 37 °C, 5% CO_2_ for 6 h. The supernatant was discarded and fresh culture medium was added. After incubation at 37 °C, 5% CO_2_ for 24–48 h until 100% confluence, the cells were harvested and marked as passage 0. Cardiac fibroblasts of passage 3–5 were used in the experiments. Cardiac fibroblasts isolated from WT and Tmem43 KI pups of p3–4 day old were treated with 10 ng/mL TGFβ in DMEM for 3 days or 7 days and subjected to immunofluorescence (IF) staining with αSMA antibody or RNA isolation.

### Immunoblotting

1–5 × 10^6^ cells were seeded, serum starved for 16 h, and stimulated with various stimulators (Human HRG (Pepro Tech, Cat# AF-100-03), LPA (Avanti, Cat# 857130C), Angiotensin II (Sigma-Aldrich, Cat# A9525) for appropriate time. The cells were then lysed in a buffer containing 50 mmol/L HEPES (pH 7.4), 250 mmol/L NaCl, 1% Nonidet P-40, 1 mmol/L EDTA, 1 mmol/L Na_3_VO_4_, 1 mmol/L NaF, 1 mmol/L PMSF and a protease inhibitor mixture (Roche Diagnostics, Mannheim, Germany). The cell lysates were subjected to SDS-PAGE separation and Western blot with specific antibodies of p65 (Santa Cruz, Cat# sc-8008), PCNA (Santa Cruz, Cat# sc-56), p-IκBα (Cell Signaling, Cat# 9246), IkBa (Santa Cruz Biotechnology, Cat# sc-371), p-p65 (Cell Signaling, Cat# 30335), α-TUBULIN (Santa Cruz, Cat# sc-8035), β-TUBULIN (Easybio, Cat# BE0025), CCPG (abcam, Cat# ab106454) and pSMAD2 (Cell Signaling, Cat# 3108). All the results have at least been repeated in 3 independent experiments.

### Preparation of nuclear extracts and electrophoretic mobility shift assay (EMSA)

1–5 × 10^6^ cells were seeded, serum starved for 16 h, stimulated with various stimulators for appropriate time, and the nuclear extracts were prepared. Nuclear extracts (5–10 μg) were incubated with ^32^P-labeled nucleotide probes of p65 or OCT1 binding substrates at room temperature for 15 min. The samples were separated on a native gel of Tris-Borate-EDTA polyacrylamide, which was subsequently dried at 80 °C for 1 h, and exposed to X-ray film.

### Serum cytokine assay

The cytokine level of serum of WT and KI mice were measured using commercial ELISA kits: mouse TNF alpha ELISA Ready-SET-Go (eBioscience, Cat# 88-7324-77) and mouse IL-6 ELISA Ready-SET-Go (eBioscience, Cat# 88-7064-88) following the manufacturers’ instructions.

### Chromatin Immunoprecipitation (ChIP)

ChIP assays were performed with ChIP-IT® Express Chromatin Immunoprecipitation Kits (Active Motif Inc.) according to the manufacturer’s manual. Briefly, 50 mg murine heart tissues were firstly minced into small pieces with blade and cross-linked in 1% formaldehyde in PBS for 10 min at room temperature. The fixation was stopped by adding glycine to a final concentration of 125 mmol/L. The tissues were then washed twice with ice-cold PBS and resuspended in lysis buffer supplemented with proteinase inhibitor cocktails (PIC) and PMSF. The lysate was transferred into a dounce homogenizer and dounced on ice for 30 strokes to release cellular nuclei. The nucleus pellets were spun down and resuspended in supplied digestion buffer supplemented with PIC and PMSF. The chromatin was sheared by adding enzymatic shearing cocktails at 37 °C for 20 min. The lysate was cleared by centrifugation (10 min, 18,000 RCF, 4 °C). Supernatant was subjected to immunoprecipitation with specific antibodies of p50 (Santa Cruz), p65 (Cell Signaling Technology) and control IgG via incubation with magnetic beads of protein G on a rotator at 4 °C for 4 h. Supernatant (10%) was used as ChIP input. After 3 times washes, immunoprecipitated chromatin was eluted with elution buffer AM2 and crosslinks were reversed in reverse cross-linking buffer at 95 °C for 10 min. The samples were sequentially used in quantity PCR assays with specific primer sets for the predicted binding sites of transcription factors on mouse genome.

### Immunofluorescence (IF) staining

Dermal fibroblasts and cardiac fibroblast cells isolated from WT and KI mice were cultured overnight (2 × 10^5^ cells/well) on coverslips in 24-well plates, rinsed with PBS, and fixed in 4% paraformaldehyde for 10 min. The cells were permeabilized with 0.25% Triton X-100 in PBS for 15 min. Cells were then blocked with 5% BSA in PBS at room temperature for 1 h and incubated overnight with anti-αSMA ((Abcam, Cat# ab5694, 1:500) in 2.5% BSA in PBS at 4 °C. After washing, slides were incubated with anti-rabbit Alexa-546 (Invitrogen; 1:600) at room temperature for 1 h. After washing with PBS again, cells were counterstained with 1 μg/mL DAPI (Beyotime; 1:5,000) for nuclei. Cells were imaged on a Zeiss LSM780 laser scanning confocal system.

### Immunoprecipitation mass spectrometry (IP-MS) analysis

We stably expressed vector control, HA-Flag-TMEM43 WT and HA-Flag-TMEM43 S358L mutant in A549 cells with lentivirus and selected the positive infected cells with blasticidin (BSD) treatment for 7 days. Three cell lines were lysed, immunoprecipitated (IP) with Flag antibody (Anti-Flag Affinity Gel, YEASEN, Cat# 20585ES08) and then HA antibody, the obtained products were performed trypsin digestion and mass spectrometry (MS) analysis. The products of every step during IP were collected and subjected to immunoblotting with Flag antibody (Abmart, Cat# M20008) to control the quality of IP. The obtained results from MS were subjected to searching and comparison in protein database bank (PDB). MS scores referred the general abundance of specific proteins detected.

### Statistical analysis

All quantified data are presented as mean ± standard deviation (SD). The 2-tailed *t* test was used for group comparisons. The differences at a value of *P* < 0.05 were considered statistically significant.

## Electronic supplementary material

Below is the link to the electronic supplementary material.

Supplementary material 1 (PDF 1240 kb)
