# Supplementary material for: TMEM43-S358L mutation enhances NF-κB-TGFβ signal cascade in arrhythmogenic right ventricular dysplasia/cardiomyopathy
Source: Protein Cell. 2018 Jul 6;10(2):104–19. doi: 10.1007/s13238-018-0563-2 (PMC6340891; doi:10.1007/s13238-018-0563-2)
Supplement: Supplementary file 1 — Supplementary material 1 (PDF 1240 kb) [file 13238_2018_563_MOESM1_ESM.pdf]

SUPPLEMENTAL MARTERIALS

Supplemental Figure 1. TMEM43 ARVD mutant elevated TGFβ signaling activity.

(A) Qiagen website predicted several Rel A(p65) binding sites on the promoter of *Tgfβ1* gene in mouse genome. (B) The hearts of WT and KI mice of 8-week old males were subjected to Chromatin Immunoprecipitation (ChIP) assays via IgG and p65 antibody. The enriched folds of antibody binding were assayed by qPCR on -11kb binding site. (C) The enriched folds of antibody binding were assayed by qPCR on -6kb binding site. (D) The enriched folds of antibody binding were assayed by qPCR on 1kb binding site. (E) The enriched folds of antibody binding were assayed by qPCR on 3kb binding site. (F) A549 cells stably expressing TMEM43 WT or S358L mutant were serum starved for overnight and stimulated with or without AngII (2 μmol/L) for the indicated time. The nuclear extracts were subjected to immunoblotting of pSMAD2 antibody and PCNA antibody.

Supplemental Figure 2. TMEM43 ARVD mutant promoted myofibroblast transformation.

(A) Electrocardiograph of littermate WT and KI mice of 6-month old males after intense running continuously for 7days. Black arrows indicated the abnormal ECG cycles. (B) The hearts of WT and KI mice of 8-week old males were fixed, sagittal or transverse sectioned in paraffin and performed hematoxylin-eosin (HE) staining. (C) Quantity PCR assays showed the mRNA level of the markers of cardiac fibroblast (*Vim, Ddr2*) and myofibroblast (*αSma, Fn1*) in the hearts of WT (n=4) and KI (n=3) mice. (D) Quantity PCR assays showed the mRNA level of desmosomal proteins in the hearts of WT (n=4) and KI (n=3) mice. Student's t-test was used for statics analysis. The star * indicated p-value < 0.05, ** indicated p-value < 0.01, *** indicated p-value < 0.005 and **** indicated p-value < 0.001 in student t-test.

Supplemental Figure 3. TMEM43 ARVD mutant did not alter fibrosis progress in kidneys.

(A) A549 cells stably expressing TMEM43 WT or S358L mutant were serum starved for overnight and stimulated with or without AngII (2 μmol/L) for the indicated time. The cytoplasmic proteins were subjected to immunoblotting of IκBα, p65 and TUBULIN antibodies. (B) The kidneys of WT (n=5) and KI (n=6) mice of 8-week old males were fixed, sectioned in paraffin and performed masson's trichrome staining. The blue staining indicated the regions of cardiac fibrosis. (C) Quantity PCR assays showed the mRNA level of the markers of fibroblast, myofibroblast and the components of TGFβ signal in the kidneys of WT (n=5) and KI (n=6) mice. Student's t-test was used for statics analysis. The star * indicated p-value < 0.05 and ** indicated p-value < 0.01 in student t-test.

Supplemental Figure 4. TMEM43 ARVD mutant accelerated fibrosis progress in dermal fibroblasts.

(A) Dermal fibroblasts isolated from WT and TMEM43 KI pups of p3-4 day old were treated with 10ng/mL TGFβ in DMEM for 3days or 7days and stained with αSMA (red) antibody. (B) Dermal fibroblasts isolated from WT and TMEM43 KI pups of p3-4 day old were treated with 10ng/mL TGFβ in DMEM for 3days or 7days. The total RNAs of the treated cells were isolated. qPCR with specific primers of *αSma, Fn1, Vim, Ddr2, Tgfβ1* and *Col1a1* were performed. *Gapdh* served as endogenous control. Student's t-test was used for statics analysis. The star * indicated p-value < 0.05; ** indicated p-value < 0.005 in student t-test; *** indicated p-value < 0.01 in student t-test and **** indicated p-value < 0.001 in student t-test.

Supplemental Figure 5. TMEM43 ARVD mutant accelerated fibrosis progress in cardiac fibroblasts.

(A) Cardiac fibroblasts isolated from WT and TMEM43 KI pups of p3-4 day old were treated with 10ng/mL TGFβ in DMEM for 3days or 7 days and stained with αSMA (red) antibody. (B) Cardiac fibroblasts isolated from WT and TMEM43 KI pups of p3-4 day old were treated with 10ng/mL TGFβ in DMEM for 3days and 7days. The total RNA of treated cells were isolated. qPCR with specific primers of *αSma, Fn1, Vim, Col1a1, Col3a1* and *Tgfβ1* were performed. *Gapdh* served as endogenous control. Student's t-test was used for statics analysis. The star * indicated p-value < 0.05; ** indicated p-value < 0.005 in student t-test; *** indicated p-value < 0.01 in student t-test and **** indicated p-value < 0.001 in student t-test.

Supplemental Figure 6. Inhibition of TGFβ signal reversed the fibrosis progress in dermal fibroblasts.

(A) Dermal fibroblasts isolated from WT and TMEM43 KI pups of p3-4 day old were treated or untreated with 10uM TGFβ inhibitor - LY2109761 for 3days and stained with αSMA (red) antibody. (B) Dermal fibroblasts isolated from WT and TMEM43 KI pups of p3-4 day old were treated or untreated with 10uM TGFβ inhibitor - LY2109761 for 2days, and the total RNA were isolated. qPCR with specific primers of *αSma, Fn1, Ddr2, Tgfβ1, Col1a1* and *Ctgf* were performed. *Gapdh* served as endogenous control. Student's t-test was used for statics analysis. The star *** indicated p-value < 0.01 in student t-test and **** indicated p-value < 0.001 in student t-test.

Supplemental Figure 7. TMEM43 ARVD mutant enhanced PPARγ co-activator expression.

(A) The paraffin sections of spleens from 129 mouse of 8-week old male challenged with 50mg/kg LPS for 6h were performed immunohistological staining via various antibodies. (B) The upper panel of Figure B indicated the procedure of IP-MS assays. The bottom data indicated the binding ability between TMEM43 and CCPG. (C) Total protein lysates from A549 cells stably expressing TMEM43 WT, S358L mutant or vector control were subjected to immunoblotting of CCPG (constitutive coactivator of PPAR-gamma-like protein 1) antibody, TMEM43 antibody and control β-TUBULIN antibody. (D) Quantity PCR assays showed the mRNA level of *Ccpg* and PPARγ

target gene - *Plin* in the hearts of WT (n=4) and KI mice. *Gapdh* served as endogenous control. Student's t-test was used for statics analysis. ** indicated p-value < 0.01.

Supplemental Table 1.

Mouse #	DOB	Sex	KI-cre	Exercise
4Vcre421	2015/8/8	M	+/-	die
4Vcre416	2015/8/8	M	+/-	run
4Vcre417	2015/8/8	M	+/-	run
4Vcre430	2015/8/8	M	+/-	run
4Vcre415	2015/8/8	M	+/-	run
4Vcre427	2015/8/8	M	+/-	run
4Vcre431	2015/8/8	M	+/-	run
4Vcre438	2015/8/8	M	+/-	run
4Vcre425	2015/8/8	M	+/+	run
4Vcre419	2015/8/8	M	+/+	run
4Vcre426	2015/8/8	M	+/+	run
4Vcre428	2015/8/8	M	+/+	run
4Vcre420	2015/8/8	M	+/+	run
4Vcre432	2015/8/8	M	+/+	run
4Vcre418	2015/8/8	M	+/-	
4Vcre423	2015/8/8	M	+/-	
4Vcre424	2015/8/8	M	+/-	
4Vcre429	2015/8/8	M	+/-	
4Vcre434	2015/8/8	M	+/-	

Supplemental Table 2.

Accession	Gene	Score A2 (Mu)	Score B2 (T43)	Score C2 (Vc)
P14923	Junction plakoglobin (JUP)	17.91	18.24	3.28
P15924	Desmoplakin (DSP)	69.37	49.22	24.05
Q99959	Plakophilin-2 (PKP2)	4.76	0.00	0.00

Supplemental Figure 1

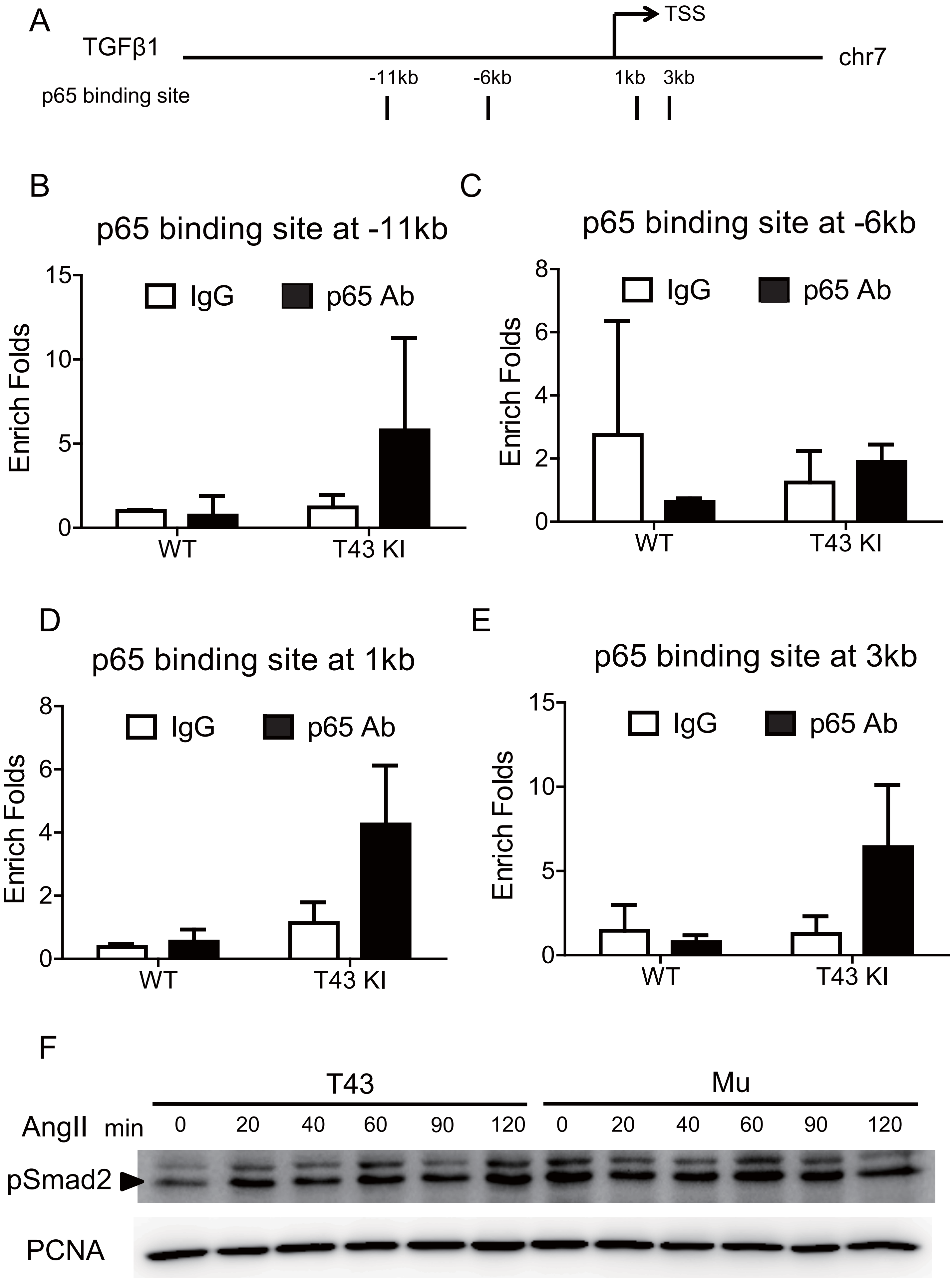

Supplemental Figure 2

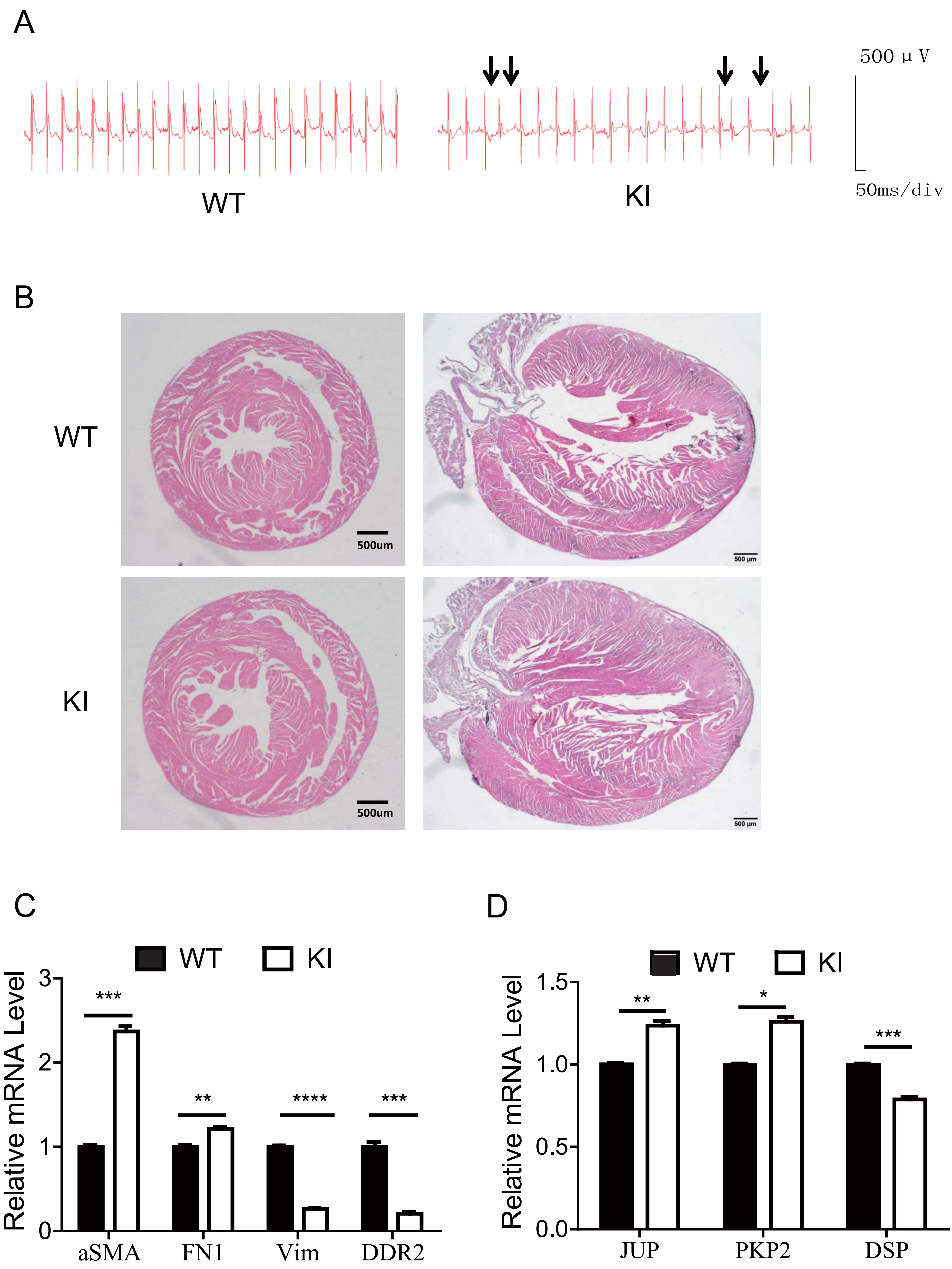

Supplemental Figure 3

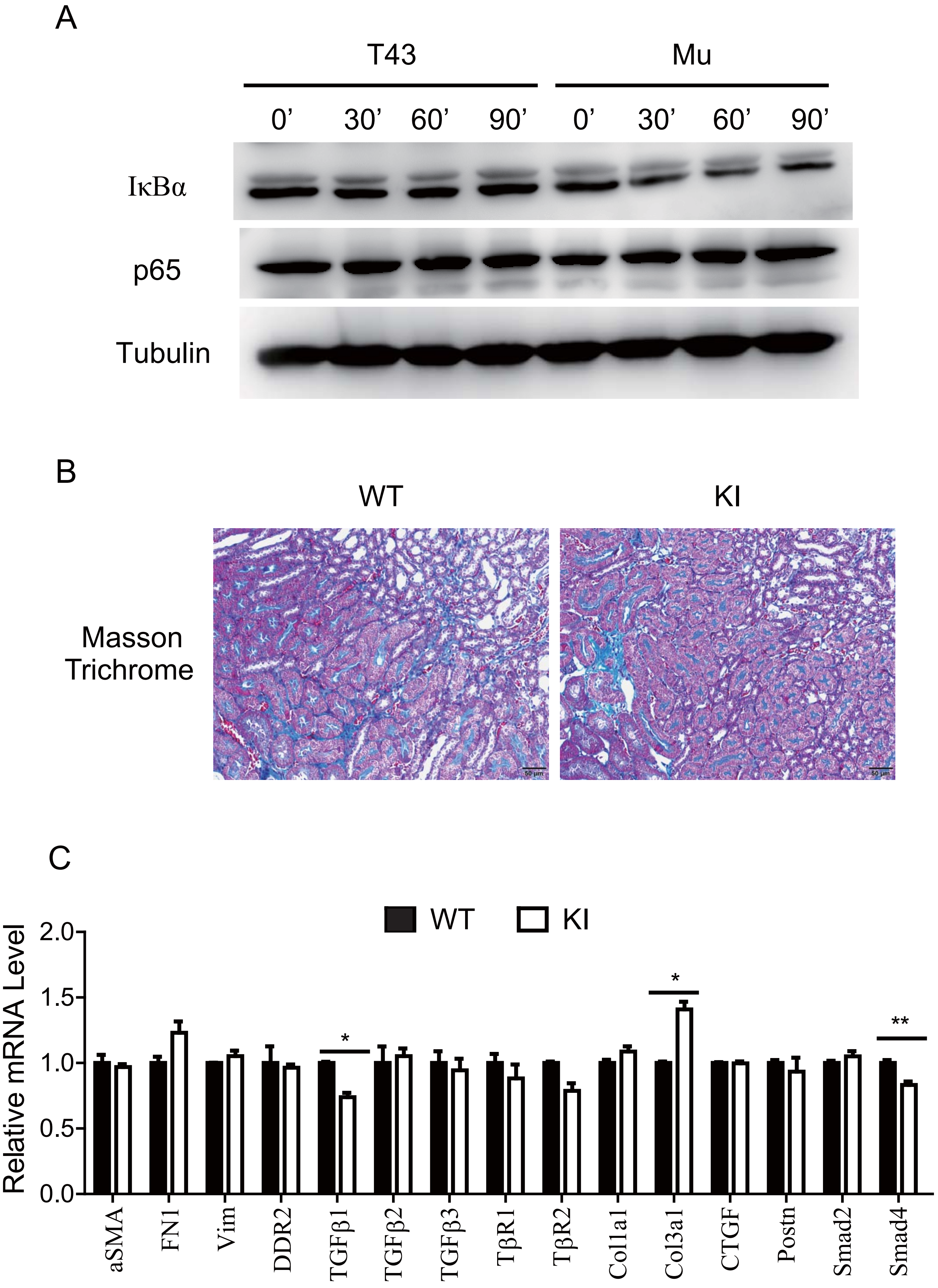

Supplemental Figure 4

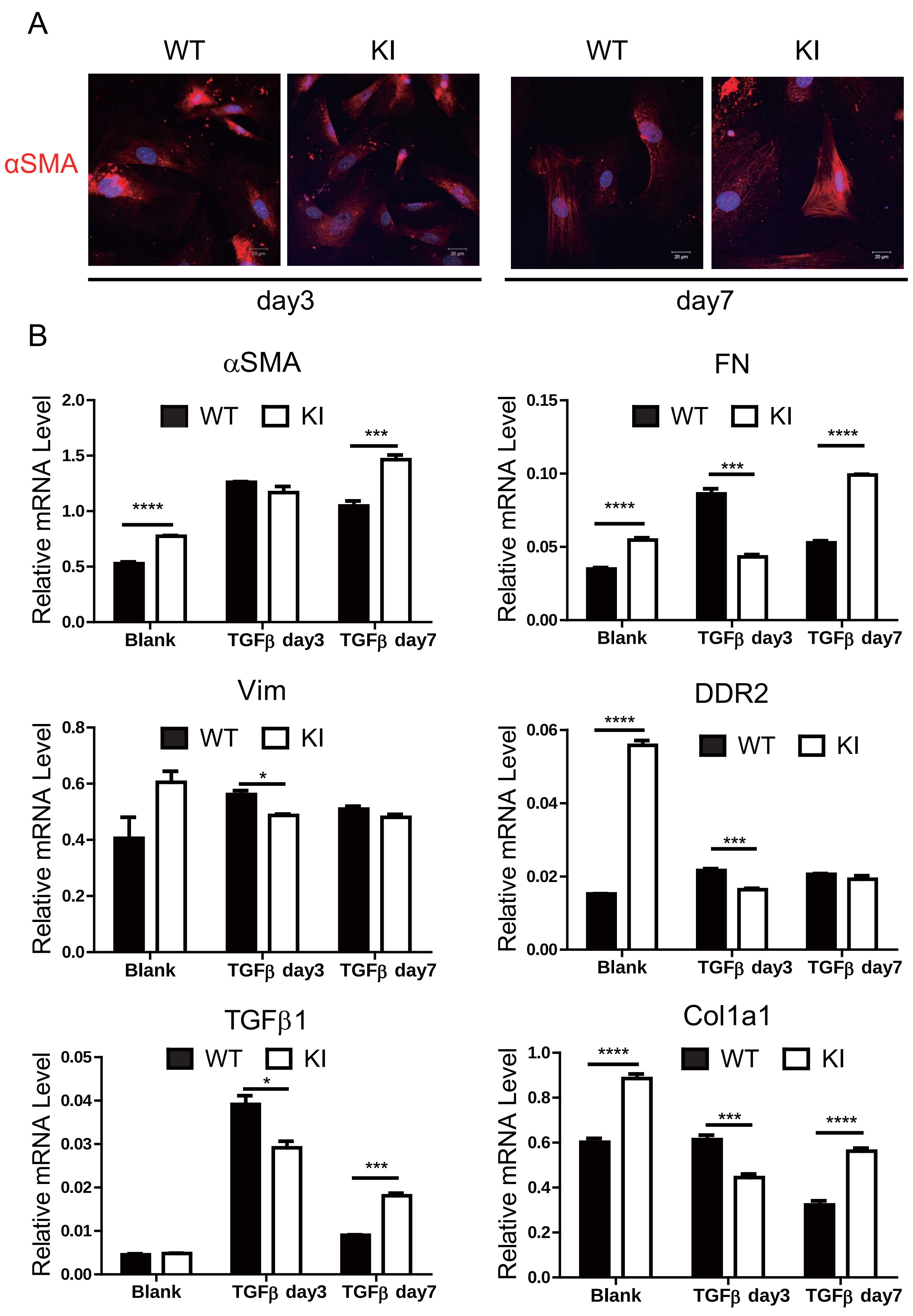

Supplemental Figure 5

A

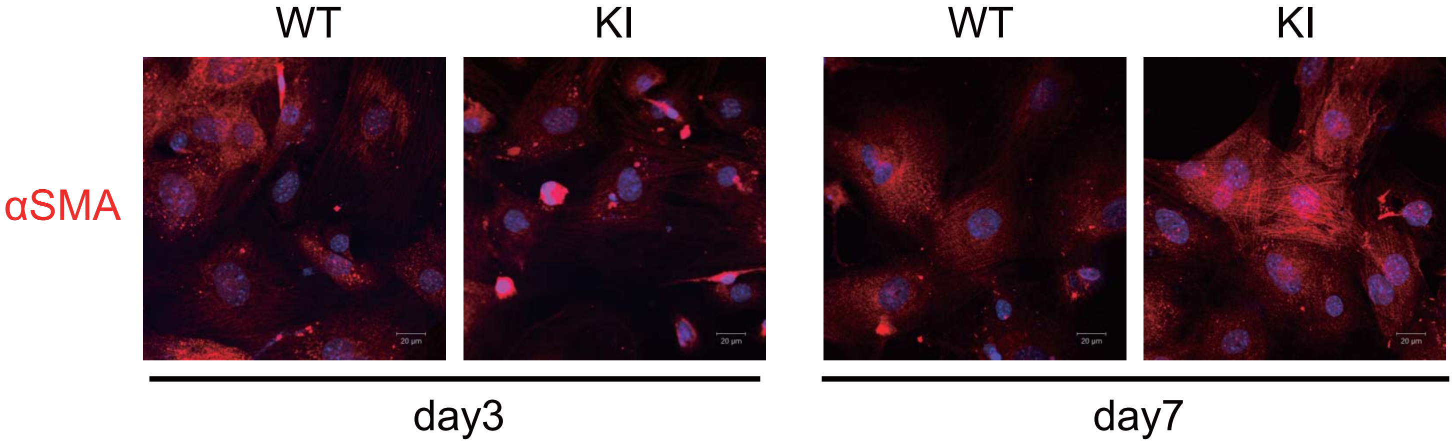

B

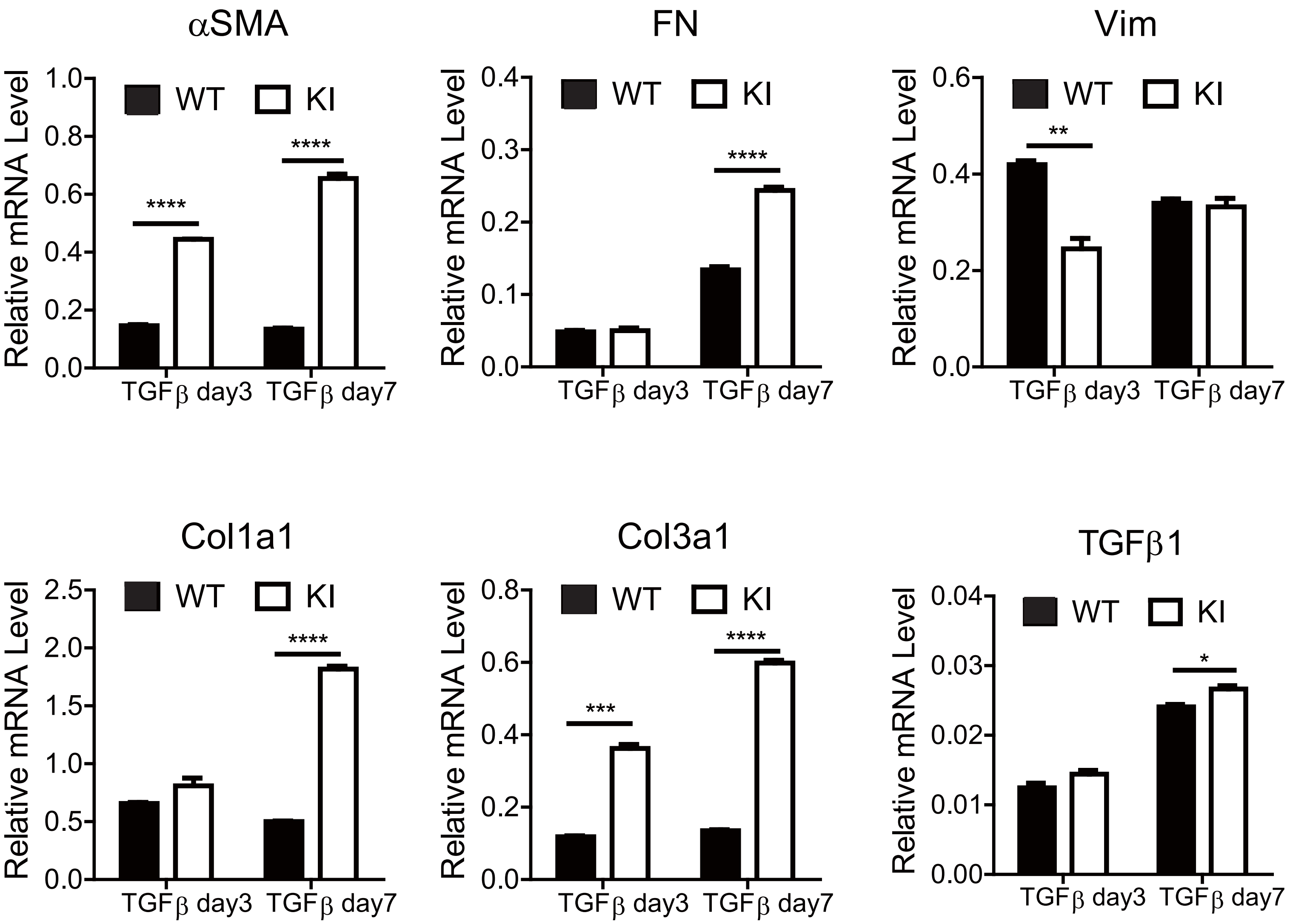

Supplemental Figure 6

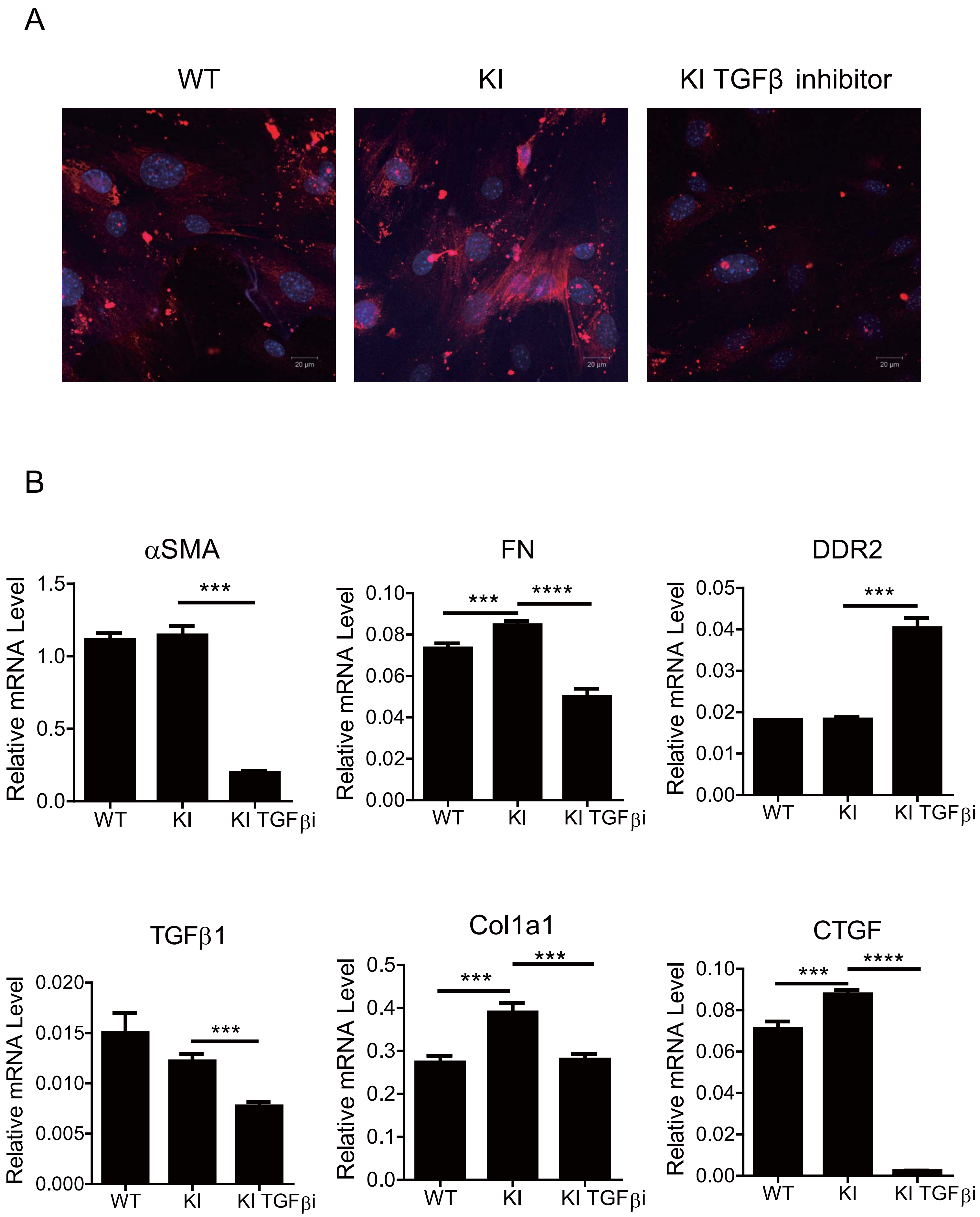

Supplemental Figure 7

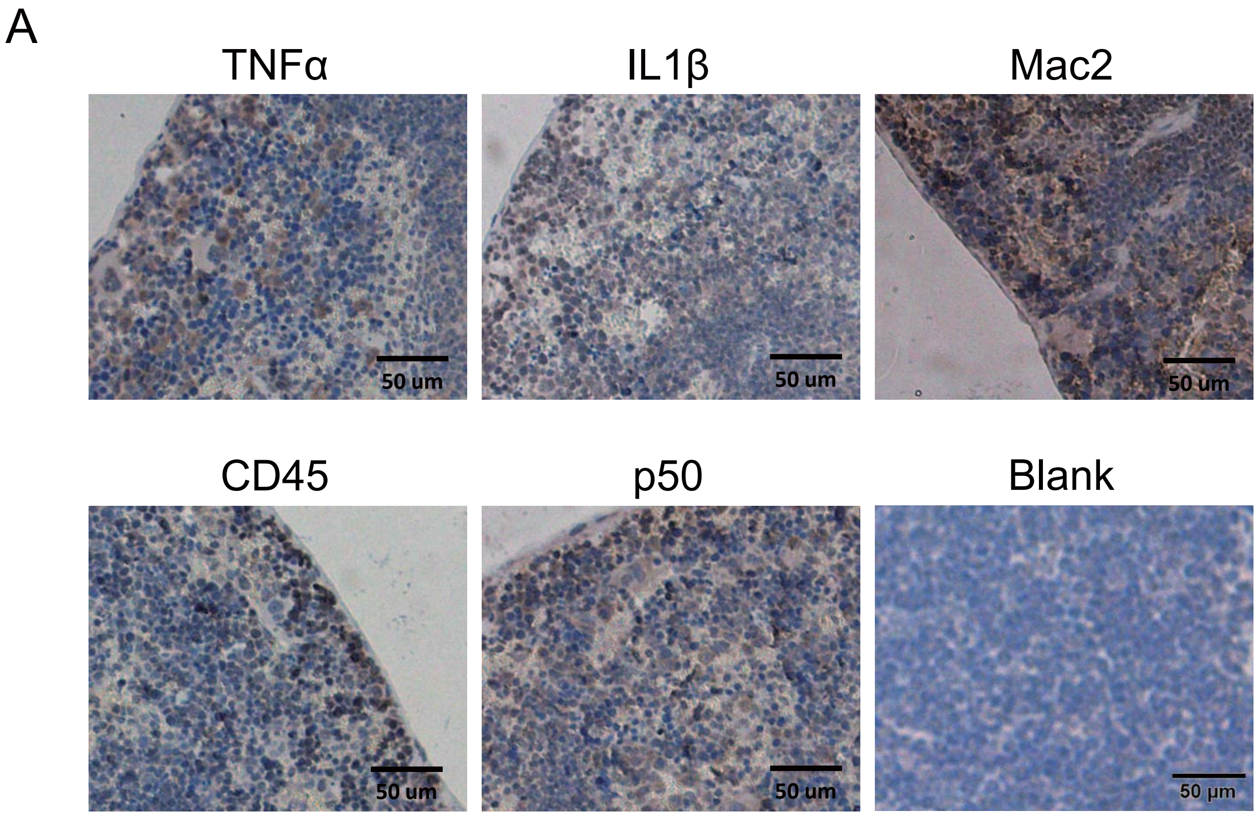

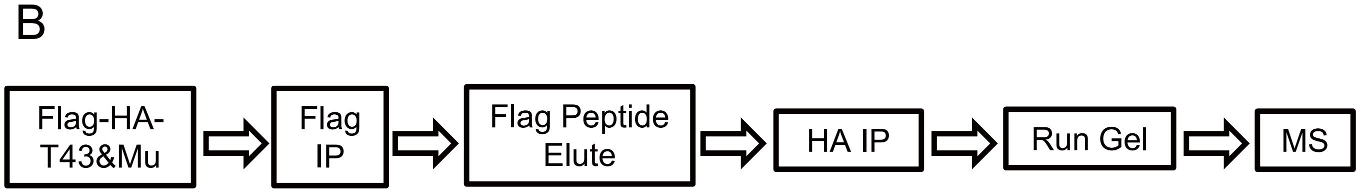

Gene Name	Mu	T43	Vc	log2(Mu/T43)
Constitutive coactivator of PPAR-gamma-like protein 1	17.79	4.11	0.00	2.113245

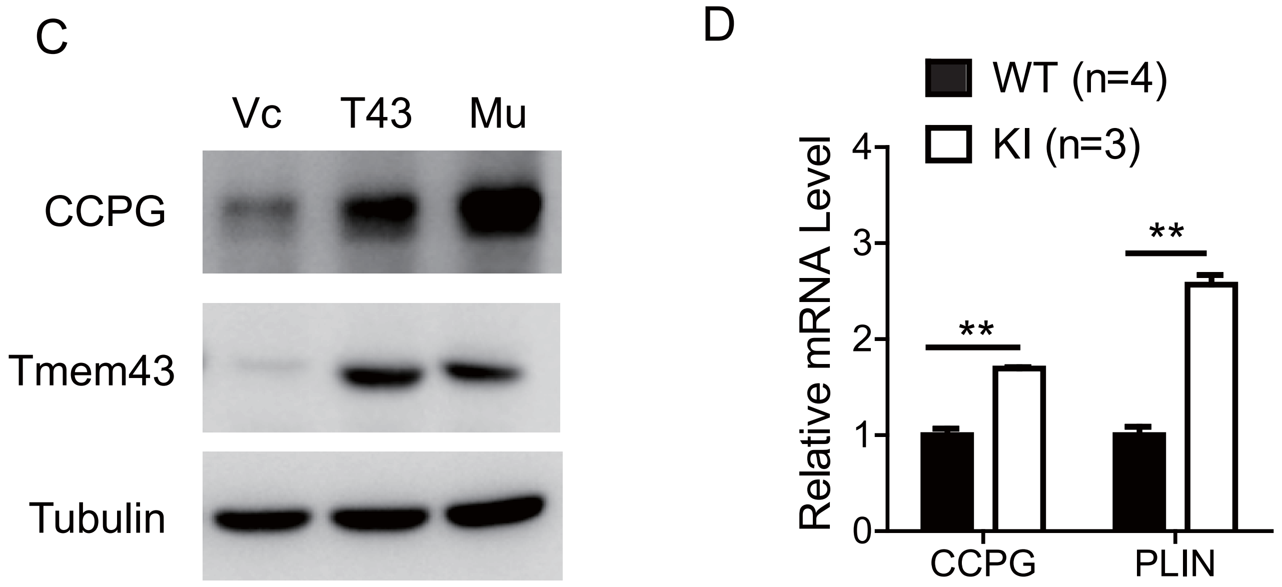

qPCR Primers

species	cell lineage	gene name	forward		reverse		length
mouse	control	GAPDH	Gapdh RT(+)	TGGCCTTCCGTGTTCCTAC	Gapdh RT(-)	GAGTTGCTGTTGAAGTCGCA	178bp
mouse	fetal heart	aMHC	aMHC RT(+)	TGGTCACCAACAACCCATACGACT	aMHC RT(-)	TGTCAGCTTGTAGACACCAGCCTT	155bp
mouse	fetal heart	βMHC	bMHC RT(+)	CCTGCGGAAGTCTGAGAAGG	bMHC RT(-)	CTCGGGACACGATCTTGGC	119bp
mouse	fetal heart	ANP(Nppa)	ANP RT(+)	TTTGGCTTCCAGGCCATATT	ANP RT(-)	CATCTTCTACCGGCATCTTCTC	117bp
mouse	fetal heart	BNP(Nppb)	BNP RT(+)	ACTCCTATCCTCTGGGAAGTC	BNP RT(-)	GCTGTCTCTGGGCCATTT	105bp
mouse	fibrosis	Col1a1	Col1a1 RT(+)	CATAAAGGGTCATCGTGGCT	Col1a1 RT(-)	TTGAGTCCGTCTTTGCCAG	111bp
mouse	fibrosis	Col3a1	mCol3a1 RT(+)	CTGTAACATGGAAACTGGGGAAA	mCol3a1 RT(-)	CCATAGCTGAACTGAAAACCACC	144bp
mouse	fibrosis	TGFβ1	TGFb1 RT(+)	CGAAGCGGACTACTATGCTAAA	TGFb1 RT(-)	TCCCGAATGTCTGACGTATTG	129bp
mouse	Adipose	C/EBPa	C/EBP RT(+)	CGCAAGAGCCGAGATAAAGC	C/EBP RT(-)	CGGTCATTGTCACTGGTCAACT	80bp
mouse	Adipose	PPARr	PPARr RT(+)	GGAAGACCACTCGCATTCCTT	PPARr RT(-)	TCGCACTTTGGTATTCTTGGAG	158BP
mouse	Adipose	Adiponectin	Adipoq RT(+)	GAGAAGGGAGAGAAAGGAGATG	Adipoq RT(-)	TGAGCGATACACATAAGCGG	108bp
mouse	NFkB target Cytokine	TNFa	TNFa RT(+)	CTACCTTGTTGCCTCCTCTTT	TNFa RT(-)	GAGCAGAGGTTCAGTGATGTAG	116bp
mouse	NFkB target Cytokine	IL6	IL6 RT(+)	TAGTCCTTCCTACCCCAATTTCC	IL6 RT(-)	TTGGTCCTTAGCCACTCCTTC	76bp
mouse	NFkB target Cytokine	IL1β	IL1b RT(+)	CTGTGACTCATGGGATGATGATG	IL1b RT(-)	CGGAGCCTGTAGTGCAGTTG	75bp
mouse	cytokine	Mac2	Mac2 RT(+)	GGAGAGGGAATGATGTTGCCT	Mac2 RT(-)	TCCTGCTTCGTGTTACACACA	85bp
mouse	TGFbeta signal	TGFb2	TGFb2 RT(+)	GTACCTTCGTGCCGTCTAATAA	TGFb2 RT(-)	GTGCCATCAATACCTGCAAATC	82bp
mouse	TGFbeta signal	TGFb3	mTGFb3 RT(+)	CCTGGCCCTGCTGAACTTG	mTGFb3 RT(-)	TTGATGTGGCCGAAGTCCAAC	75bp
mouse	TGFbeta signal	Tgfbr1	Tgfbr1 RT(+)	TCTGCATTGCACTTATGCTGA	Tgfbr1 RT(-)	AAAGGGCGATCTAGTGATGGA	100bp
mouse	TGFbeta signal	Tgfbr2	Tgfbr2 RT(+)	AACATGGAAGAGTGCAACGAT	Tgfbr2 RT(-)	CGTCACTTGGATAATGACCAACA	90bp
mouse	TGFbeta signal	Smad2	mSmad2 RT(+)	TCCGTACCACTACCAGAGAGT	mSmad2 RT(-)	GGCGGCAGTTCTGTTAGAATC	87bp
mouse	TGFbeta signal	Smad4	mSmad4 RT(+)	ACACCAACAAGTAACGATGCC	mSmad4 RT(-)	GCAAAGGTTTCACTTTCCCCA	83bp
mouse	TGFbeta signal	periostin	Postn RT(+)	GGTGTCCTAGAAAGGATCATGG	Postn RT(-)	CAGAGCACTGGAGGGTATTTAG	85bp
mouse	TGFbeta signal	CTGF	CTGF RT(+)	ACCTGTGCCTGCCATTAC	CTGF RT(-)	GTCCCTTACTTCCTGGCTTTAC	105bp
mouse	TGFbeta signal	Fibronectin	mFn1 RT(+)	GATGTCCGAACAGCTATTTACCA	mFn1 RT(-)	CCTTGCGACTTCAGCCACT	119bp

Mouse TGFbeta1 genome: -6kb promoter

NFkB(p50) binding site: gggACTTTCC; gggRNNYYCC, R=purine AG, Y=pyrimidine CT

cctacgggcaagggtaaagcggccctacacccgagacgagacgcatgcgccttttgaagc
ttggctgatctgccgcgaaggggcggggccttgcacatgtgggcggggccacaaccctgg
ggcggggctttctaagcgtctgagccttggtttatttattggtgcaggttcccacacctc
tgggagacttccccattttcttgggcttttctggggacgtggcttgtatctgtgatgaa
ggccacattgtcagtgggttggtggaactagcttagaatcacagtgctgttctttatgtg
ataggcttcagttttcccagctgtcagatggaaataatacagttacctcacagggttgtg
attgaattttcctttcctagagccatggctgaagtgcacgtgatcgggcagatcatagg
ggccaccggtttctcggaaagcagcctcttttgcaagtggggcatccacacaggtatcct
cctcacaccttgttcatctcccaaacaatacccagctccctcccctagcctaagtctcca
tgttccttcctcgattctccaatgaactcatactgtttgcttctctttatccttgtcttt
catgttatctcaccatcggaaaacatagcccctaagcatatggggctgtgacatagaaaa
cacgcgcgcacacacacacacacacacacacacacacacacacacttcctgtcctcaggc
cagtagacaaactaggtgtgcttatgcatatctgtaattccagcacctaggaagccttct
attacaagtttggggccctgtctcaaagaaaagagcacaaaatatatacaagattattgt
aagttctagggaaaaaaagaaagtgatgtgctgggggctgatttgggatgtggaggtctg
agatcagagaaagcctttctgtgcaagtggcacttgaattgggggcaggaggaaaacag
ccggaaatgggcacccagtagcagacgtttcagggcaaaaggaacagccgctgccaaggc
cttgaggcaagggaacaggatgggaccagtgaggcttgagggtggggagcggcagtggca
gatgagtgacaggatccaggtcacacatgggccttcgtggcaacagtgaggagtctgaaa
gcttcgtgtgtgtgtgtgtgtgtgtgtgtgtgtgtgtgtgtgtgtgtgtgtgtgttggga
gggggcagttagaatcccagagagcagataaggtgaaggcattgcccagagcgtttagca
gagcatctggggtgtagtgctcagtatgtgggcactgcttctccttgctcctcactgggg
ttgtgatgtgctcgcagatggtgcccagtctcttcttggtctttgacggaggcttctgaa
tgtggggtgcatagaggaaggaaaatgctggggatctcgtcacccggggagcgtaggtca
tccttagctacgacagaggtttgaggctagccttggctatatgagaccttgtcttaagaa
aggaggctctctgttggtgctgagagtgcagctcatcagcagagcatagagcgtgtgcct
ctggatcacatcgccagctgcagtcatgaggattaacaagtgaataatattttaaaaatt
catagttcatccttgctatgtgagtcctctcataacttgtgagattgtgtttttttgtcgc
ttgttttatagacagggaaacagagagaagctactctgccagggttgcatcaacttgggc
tggggagtgtagactgttgctagagagctcttgcatatgtactgctctaggttccatccc
taacactgcaaaatacagaacacatatatgaatcgtgccaggatttctacacgggattct
ctgtgtagccctggcagccctgggaactccttctgtagaccaggctggctttgaagtcac
agagatcagcctgcctctgcctccccagggctaagaccaactgtgtgcaccaccactgcc
tggctttattctctctcacgtgctcttcaacccctccaatttttctttagagactgagac
ccacattgtagcccatgctgacctgacactcatcaggcagcccaagaaagccccaaacaa
actcccaagtgctgagatttcagatgtacactgccagtgccaagatctggatccagacac
ttggctctagagtctacactgtgtaactcccactcccagataccagagggaacaaactca
ttgccacggcaggtccccttcttgaaggttgctatcccccaaaccctgatgtcctggttc
tcattacaggggccgcatggaagctcctctcaggtgtacgagaaggccagacacaggtag
acacaccccagataggggacatggcttactggtcccaccccattgacctgcacttcgcta
ctaaaggtctccaaggtgggtacaggatgacaggatggggaggggcagtcaaactaagag
gagctgggcttggaacaccatggctgtctgctccctgtactttgtgggtcccttttctgg

tacagcctcccctagagcagtgcatttgaggcctcacccgagctattcagaaccctaggc
ctgtggaatgtgccggaggttggggtagagaagatatcaggaagtgggggtcaagtggga
ggggcctttgactaacggccactgaggaggctgcatagggtaaagagagatagataggga
ggagagggacaggaaatgggccagttgccgttgctcacacaggcagaggttctgctcaaa
ggggaagtgagtcttcgagggaggaggagggtatcatgtcagtgcagcttttctggttaa
aacacagcagattctgggaatgaggactgttgactgagcctacgtctgtccagcttgagc
agggacatccccaactaagaaggaccctccaagccctgtcatcaagggttccattatagg
tagaagatagacctgtttccaaatgtggccagcacagaggccctggaaacacagaagtca
tctctgattggtcccatggccttatagggcagagcagttagagttctgctgtaaagggcc
aagagaagctaactaggcccaaggacaaagacagagggtggagtcagagagctggagcca
ggtagaaatggggaggtgtgtcctgggtattcgctgtctgtgggtgagaaggacagtggg
ggatgaggcggcagggacagaatgtaaggcctcgcctgcaaaggcactaagactgattct
cgaaggctctggggagcccaagaagaaatggatggatcagattctccttctgaacaattg
tactagtttgtgagggtgatacgttggaagttttcctcagtcctggccttagctgtcttc
ctccctgtttgcttccttcttagtctctcagctcaccaacaaaccctggcggctctgaaa
cccactttgtttctcaccccatcccacccagccttctctgtccatcttctctttccggg
accatttttgtttttttgttttgttttgttttgttttgtttttcaagacagggtttctct
gtatagccctggctgtcctggaactcactctatagaccaggctggcctcaaactcagaaa
tctgcctgcctctgcctcccaagtgctgggataaaaggcgtgtgccaccacgcccagcct
tctgggaccattctttctttctgtcttcactgtgctctcagggttggatactggactgga
ccctgggaggacctaaatacagcatgttcttcctctgtatggaacctgcctgcaggcctc
ctatcgctcaagagaaaagccggagccttctctggcgtcacaggcctatggaatctggct
gtcacctctgtcctttgttccccacgactccccgttctagcctcattggccccaccaggc
tagctcctacttcggaacttttgcactgactggatctgtcattcccctaggtgcctagag
agcccacttctttaagcatttatgttgcatcctggttggcttgtttgctttttgagatag
agtctcagaacatagtccagcctagcctggggcctcagcagcccaagtgtgtgccaaaat
gtcacccttcagatatgtagccctggttgtcctagaactcattgtgtagcccaggctagc
cttgaacttctggggatcctcttgcctctacctcccaagtgctggcattccagctgtaca
ctgcagtgttcagccccaaatgtcaccttctttaggaaagcttccttcagctgctcccca
attttccaaactacatctccacccctccctttctggctgttgtttttccccagagcggtt
atcaccatatgacataccctataatttacttatttactatgttggttgtctaggatgtca
gcttcacggggcaggaatttttgtctgctgtgttcattgctgtgtccccagcctggagaa
cagggcctgccaggggagggcttagggttgggggggggggggctgggctgcatctccaa
gcattggactgtcaggctgggacactcagctgtgcattgaggccactagaaacctaacga
aggggagagatggctccactggggaagaaaggaaatctgagtcaggtgcgtgagagcaaa
gcaagtccatgaggaggagggcggaaggggtctaagctgaggtgctgacctgggggcctg
ggagagtgaagagaccacaaagggaggtcagacgaggccagacttgacttgagaggttgg
actttgttctgtggcccaggggagtcatgggagggttttgagcaagagggtaatgtggat
tgctctagattcctgtgactactatgtggagtgagtgggagatgagaacccagagaggaa
attcaggcaataatccaaggtaaaagtactgtgcgaggtctccagtggtttcaatgctgg
gaacccgggagcagggttctgcccctttgtccttttctctaacgcctctcctcccgcagg
ttggcctcgactccatctccaggtgtggtcccaggacagctttggccgctgccagcttgc
tggctatggcttttgccatgtgcctagcagcccaggcactcatcagctggactgccctac
atggaggcccctgggcagttggagggagcagctagcacgggctttcgtgggtggtgggcc

acagctgctgcacgcagataccatctacagcggggctgaccgctaccgcctgcacacggc
cgcgggtggcacagtgcaccttggtatcggcctgctgctgcgccacttcgatcgctatgg
agtggagtgttgagggacttcattgctactaatcgccatcatccactgagcacataatgg
cacagcagtgaagcaggcacgtggactctggagactgtcagtcctgatcacccccaagtc
atggcccctatgccatggactgcaaggccaggaactcccctgtggcccatgctccactga
actacggggtagaaagggctgtgggttggagggacatgagcagggcccactgtttggact
gtcgggggcacttaataaaagcgtgtatttctcatgggtaaggtgcctccttgtatccgc
taaagctctcagacgtcggtctccttatttcactctccttgacactctcatccgcaaagt
ctgcccctgcctttcgctctccaccgcctaggtccccacttctaatacaggactctgttt
tctcacgaagtcccaatttttcctttgacccttcaacaactcccaaatccaccggggcgc
tttggttggtcaccggctttagtagtgctccgccgccaggaggcagcaccctggaggtgg
ggcgaggccggggtgcccgccccctccccgcagggctgaagggacccccttggagcccg
cccacgctaagatgaagacagtgccccccatgccctcccctggggctgcccccgcccc
gcgcgcgcttcctgggtggggccggggcggcttcaaaccccgccgccccagccggtcc
c

ChIP qPCR Primer:
6k-1F: CTGATCTGCCGCGAAGG
6k-1R: TGGGAACCTGCACCAATAAA 110bp
6k-2F: GAGACGCATGCGCCTTTTG
6k-2R: ATAAACCAAGGCTCAGACGC 118bp
6k-3F: <u>gggccttgcacatgtggg</u>
6k-3R: caagaaaaatggggaagtctc 119bp

-17kb promoter:
tttttttttttttttttttttttgtcttaagccagcaccctcccagtcccaaacacagactaaccttgt
gggccagacagaaggcagcgatgcccacaggccaaaaacaacacagcacagagaataccgccaggccga
ggtggtcatggggaagaagtggagagagccggtcgcccccatcatcatctgagtcactgtcactggata
atgtgtcctgcagataggagagagc<u>acaggtggtggtcctca</u>cccacctccccatcgcccccgtagacc
agtgcttccctggggccttcccagagactttctgtcccctagatcccttgaaggggcgacagtggcct
gactgagggtctccagagagacccacccccaggtgagttcattctcaagggtccaactatgggaagatt
gctcataatccctgagtgaagacatctgcagtgggggcaggctttgccttgggatgagaacctgta

qPCR Primer:
17k-1F: AGATAGGAGAGAGCACAGGT
17k-1R: CTCTGGAGACCCTCAGTCA 145bp
17k-2F: CCGTAGACCAGTGCTTCC
17k-2R: GGGTGGGTCTCTCTGGA 107bp
17k-3F: ACAGGTGGTGGTCCTCA
17k-1R/3R: CTCTGGAGACCCTCAGTCA 132bp

Mouse TGFb1 genome: 1kb and 3kb promoter RelA(p65) binding sites

cgccgccgccgccgcccttcgcgccccaggccgtccccctcctcctcccgccgcggatcctccagacagccaggcccccggccggggcagg
ggggacgccccttcggggcacccccggctctgagccgcactcggagtcggcctccgctgggagccggcaaaggagcagccgaggagccgt
ccgaggccccagagtctgagaccagccgccgccgcagggaggaggggggaggaggagtgggaggagggacgagctggttgagagaaga
ggaaaaaaagttttgagacttttccgctgctactgcaagtcagagacgtggggacttcttggcactgcgctgtctcgcaaggaggcaggacctg
aggactccagacagccctgctcaccgtcgtggacactcgatcgctacccggcgttcctcagacgcccctattccggaccagccctcgggagc
cacaaaccccgcctcccgcgaagacttcaccccaaagctggggcgcaccccttgcacgccgccctccccccagcctgcctcttgagtccctc
gcatcccaggaccctctctcccccgagaggcagatctccctcggacctgctggcagtagctccccctatttaagaacacccacttttggatctca
gagagcgctcatctcgatttttaccctggtggtatactgagacaccttggtgtcagagcctcaccgcgactcctgctgctttctccctcaacctc
aaattattcaggactatcacctacctttccttgggagaccccaccccacaagccctgcaggggcggggcctccgcatcccacctttgccgagg
gttcccgctctccgaagtgccgtggggcgccgcctcccccatgccgccctcggggctgcggctactgccgcttctgctcccactcccgtggctt
ctagtgctgacgcccgggaggccagccgcgggactctccacctgcaagaccatcgacatggagctggtgaaacggaagcgcatcgaagcc
atccgtggccagatcctgtccaaactaaggctcgccagtcccccaagccaggggggaggtaccgcccggcccgctgcccgaggcggtgctcg
ctttgtacaacagcacccgcgaccgggtggcaggcgagagcgccgacccagagccggagcccgaagcggactactatgctaaagaggtca
cccgcgtgctaatggtggaccgcaacaacggtgagctccgaggggccggggagccaggagggagcccccagggggcgccggagtgcag
gggtcaccgggaggaaattaccctcagaggaaactggctggaggaaggggagccctgggggcaccgggacaactgtgtgggtgtcccaa
agaggctggcactttcccgtaccccaggtgtctttgagaggaatagacagcctgccaaagtgatcctctcctagaaaagcaaggtcgtgggg
agagtcccctctagaagaggagcatttggcaggttttgttttaagttcccctaatggtgcctaaaaatgcagggctcgctgggtggtctctttcc
agaaaggtagaacccagctcaagagaatggaaggtgtccaacatttcggaatggagatgcaatgtggaaagccgaccccagaccgggtcg
tgagatggagagaaaacctaggggatgggtaccctagaactgccaaataaaatctctagtgggcttactctcagaaagcgacaggcgcac
gctagagagtgtgatctgtggagtcactacggggttacaagtgaaaaaggaaagcgacgttgttgggagaaggactctagagaggagcag
agagggtgcggaaaatgagcccggaggtgtcacgagtgttttgcttgggggcgtaaaagagcgcgcgagccggcaggcggagcccagtg
aaggaagcgagcgagtcctcaagaaatctagaatactgggtcattgggaaggagtcataaaaaggggagactggcttaggaaggtggcc
ccccgcggcagccaggcagacggagcagggataccatgccatccaggagtgagtggcgagcgcccctctcctgggcgtggaagggagtg
gctgagcctctggagtcgagccctagaggagcagctagaagtaggggagaggcagtcttcaaaactcaaatgaaaaaaccgaatcggtaa
ggatgaggaagcccccacgtgggtgccacgcgcgtggggaggagcttgcgcttcttggggggggtaagaggaccccacggaactgaacgct
gacaggctgtaaccccaaggtcccagttcctcctaccggaggctggggaaaacccagcgtccggtcgccacgccctcctcccttccctttcct
tcccgtgcccgggaggaggggagacagagatcgcgcgcggagcctggggcgagacgaggcaggtccggatcccgccctccctaggctgtt
agtctcccgccctcctcaccccagtagtgggcggggaaactcacggccccgccctgtaggcgcccacgcgggacccaagcgtcgtgtgtga
ccggggaactttaggtgtcagtagcttctccaggtttcaccttcctaaccccagaggtggacttaaggattatttgcacattgctggacccaatt
gtggctcaggcctaatgacacccctccctagccacgacttaatgtgacctcttcgttggagaagtcctttactctcctgagactgtatttcttacg
catatatctcttccggcatgtgcctctcatttccgtctcctctcccacatacctattcatctctatgtcaccccatttccctctgcgacacccacaat
ccttcctgccctcctatctctcccctatcggctacccccagctgagatcccatccctccattgtggtttgctgagtaacaaccgaaccaagaata
gaggccctctgtccttcatcgacgtctgggatcaccatctcatccccactacaagaagccagaagccccagcagacgcagaatttcgggattt
tgccccttcgttccccagtgcagacatcctttaggctaggaagccctttagtgaatggggctcccaaacctgcagtcactcttacctagaaatc
ctctttgctgctaacacgaccaggaaatgggctaaagaggggagtaactaatgacactgatgacaggcaaatgataataaacccccactgg
ccaggaaatggcagagttttgggattaggaccactgtctggttgtctccatctgatcccccccctcccctgcttatcctcattcccactcagttctc
cccctccatccacctcccatgacttactgactcgctgtgcaggggtggattggtacggggtgcgggcttccccttctcaggagaggagggggt
gagggaaggggatgtgaggggggactggaaggagggaaagctgaagtcgggttataaagtgaatgaatgaatgaatgaatgaatgaac
gaacgaacaaacaaataaatgggggggggcaacccaatcccaggctgccaagtctgtctatgactgtagtcccagtactggggagacaaa
ggcaaaaggactgcttcaagacca

p65 1kb F1: ACTCCCGTGGCTTCTAGT

p65 1kb R1: CCTTAGTTTGGACAGGATCTGG 133bp

p65 1kb F2: CTACTGCCGCTTCTGCTC

p65 1kb R2: GCTCCATGTCGATGGTCTT 97bp
p65 1kb F3: GTGGCTTCTAGTGCTGACG
p65 1kb R3: TTCGATGCGCTTCCGTTT 94bp

p65 3kb F1: ACGGAACTGAACGCTGAC
p65 3kb R1: AAGGAAAGGGAAGGGAGGA 109bp
p65 3kb F2: AGGTCCCAGTTCCTCCTAC
p65 3kb R2: GGGCACGGGAAGGAAAG 86bp
p65 3kb F3: TTCCTCCTACCGGAGGCT
p65 3kb R3: GCGCGCGATCTCTGTCT 105bp

Moues TGFb1 -6kb promoter: RelA(p65) binding sites
Cctacgggcaagggtaaagcggccctacacccgagacgagacgcatgcgccttttgaagcttggctgatctgccgcgaaggggcggggcc
ttgcacatgtgggcggggccacaaccctggggcggggctttctaagcgtctgagccttggtttatttattggtgcaggttcccacacctctggg
agacttccccatttttcttgggcttttctggggacgtggcttgtatctgtgatgaaggccacattgtcagtgggttggtggaactagcttagaatc
acagtgctgttctttatgtgataggcttcagttttcccagctgtcagatggaaataatacagttacctcacagggttgtgattgaatttttcctttc
ctagagccatggctgaagtgcacgtgatcgggcagatcataggggccaccggtttctcggaaagcagcctcttttgcaagtggggcatccac
acaggtatcctcctcacaccttgttcatctcccaaacaatacccagctccctcccctagcctaagtctccatgttccttcctcgattctccaatga
actcatactgtttgcttctctttatccttgtctttcatgttatctcaccatcggaaaacatagcccctaagcatatggggctgtgacatagaaaac
acgcgcgcacacacacacacacacacacacacacacacacacttcctgtcctcaggccagtagacaaactaggtgtgcttatgcatatct
gtaattccagcacctaggaagccttctattacaagtttggggccctgtctcaaagaaaagagcacaaaatatatacaagattattgtaagttc
tagggaaaaaaagaaagtgatgtgctgggggctgatttgggatgtggaggtctgagatcagagaaagcctttctgtgcaagtggcacttga
attgggggcaggaggaaaacagccggaaatgggcacccagtagcagacgtttcagggcaaaag

p65 6kb F1: CTGATCTGCCGCGAAGG
p65 6kb R1: TGGGAACCTGCACCAATAAA 110bp
p65 6kb F2: CCTACGGGCAAGGGTAAAG
p65 6kb R2: CAAGGCTCAGACGCTTAGAA 149bp
p65 6kb F1/p65 6kb R2 85bp

Moues TGFb1 -11kb promoter: RelA(p65) binding sites
catccttcagtgacgttggtggttggtggtgtccagggttgggctctctgcatacagctcctttgcaggcattccaaggcactcgatgtgtgaac
tttggaccatagatagtgtcatctctctctaaaccgaattccttcatctggaaactcgggacatatgcccatcctcctagatgtgataacaatta
aacatttttttatgccgagtgtggtggcacacgcctttaatcccagcactcgggaggcagaggcaggcggatttctgagtttgaggccagcct
ggtctacaaagtgagttccaggacagccagggctacacagagaaaccctgtctccaaaaacaaaaacaaaaaaatttttatattacatttat
ttcttttgtgtggtgcacacatgctatgtagagcaagcatgtgtagaagtcagtggatgaattgtaggagttggttcttttcttccaccagatatg
ttcctgggatccaacatagattatcaggattagcagcaagcccccctttcctctgagtcatttccacagcccgacatatggattttaacttagat
gtgacaacaaatgcccacacatttacccaaagtctggcaaagtaagcgcagtgaacacatgtaatgtgtttatcaattttatgatttataatac
gtgtccgttcaactgaagaacgctcctggccctccaagacatcccctccttcaggaagcccacccagtttgcactgcctccctttcctgccgcc
ctccatctggctagacaggaggattcccgatgtgaggccgtggagttccctagctctagggtgccagggaactttgccaagagtccaaacac
tcaagctggcaccggccccactcccggagagagcagatggcgtctgggagcatgcttggctcagtctgagtgtctggcacagagacccacg
ggagggataacgccaggggcaagggccaggatggttttcaccagaaggtatttactagctccttcctcttgctctatttgtcttttacgccacgt
taaaaaactgctacccgcttccgtttgcatgccgctcctgcctgctcccatcaacaccagctccactggggctgggatttagtctcttttgttca

gggatgtgcctcgggcctgaaaaaaaaagtgcatggcacaaagaaggccctcctttcggactcacggatgagggtatgaactcctaactagc
ctcatttttccatcagaccactgagctcagtaacacccatcatccttaccctcctagtgaattcctgtcaagtttgaactatggcctccatagtta
gccttgtggagtctccccagcccacctctcccagcaggcctggagtcttattttttattttgtgacaggcctagaatgtcaagggggggtgcgtg
gctagagaatgagaatccaggacttcctgcatgctaataggcaactgcatctgcacgttagtcacttcctcaacacctcactggggaattcta
ggcagaggctctaccactgagccacacccccagcccctcactctgtagtctatgtcccataacatgatgcccaaaaatacagtatcacaccta
tgcttttcctgatatatcattcagatccaatcataacaattatgagccgggcgtggtggcgcgcgcctttaatcccagcactagggaggcaga
ggcaggcggatttctgagttcaaggccagcctggtctacagagtgagttccaggacagccagaactatacagagaaaccctgtctcgaa

p65 11kb F1: TCTGGCTAGACAGGAGGATT
p65 11kb R1: AGCTTGAGTGTTTGGACTCTT 94bp
p65 11kb F2: TTTGCACTGCCTCCCTTT
p65 11kb R2: GACTCTTGGCAAAGTTCCCT 112bp
p65 11kb F3: CAGGAGGATTCCCGATGTG
p65 11kb R3: GTGCCAGCTTGAGTGTTTG 89bp
